# Histone Demethylase JMJD2B Functions as a Co-Factor of Estrogen Receptor in Breast Cancer Proliferation and Mammary Gland Development

**DOI:** 10.1371/journal.pone.0017830

**Published:** 2011-03-18

**Authors:** Masahito Kawazu, Kayoko Saso, Kit I. Tong, Tracy McQuire, Kouichiro Goto, Dong-Ok Son, Andrew Wakeham, Makoto Miyagishi, Tak W. Mak, Hitoshi Okada

**Affiliations:** 1 The Campbell Family Cancer Research Institute, Ontario Cancer Institute, University Health Network, Toronto, Ontario, Canada; 2 Biomedical Research Institute, National Institute of Advanced Industrial Science and Technology, Ibaragi, Japan; 3 Department of Medical Biophysics, University of Toronto, Toronto, Ontario, Canada; 4 Department of Immunology, University of Toronto, Toronto, Ontario, Canada; Massachusetts General Hospital, United States of America

## Abstract

Estrogen is a key regulator of normal function of female reproductive system and plays a pivotal role in the development and progression of breast cancer. Here, we demonstrate that JMJD2B (also known as KDM4B) constitutes a key component of the estrogen signaling pathway. JMJD2B is expressed in a high proportion of human breast tumors, and that expression levels significantly correlate with estrogen receptor (ER) positivity. In addition, 17-beta-estradiol (E2) induces JMJD2B expression in an ERα dependent manner. JMJD2B interacts with ERα and components of the SWI/SNF-B chromatin remodeling complex. JMJD2B is recruited to ERα target sites, demethylates H3K9me3 and facilitates transcription of ER responsive genes including *MYB*, *MYC* and *CCND1*. As a consequence, knockdown of JMJD2B severely impairs estrogen-induced cell proliferation and the tumor formation capacity of breast cancer cells. Furthermore, *Jmjd2b*-deletion in mammary epithelial cells exhibits delayed mammary gland development in female mice. Taken together, these findings suggest an essential role for JMJD2B in the estrogen signaling, and identify JMJD2B as a potential therapeutic target in breast cancer.

## Introduction

Estrogen plays an important role in normal physiology and several human diseases including breast cancer. The estrogen signaling pathway is a reliable therapeutic target for estrogen-receptor (ER) positive subtype of breast cancer. Understanding of how ER regulates transcription is key to overcoming resistance to existing selective ER modulators (SERMs) and identifying non-SERM targets suitable for novel therapeutic approaches [1].

The biological functions of estrogen are mediated through ER, which regulates transcription of ER target genes by binding to estrogen responsive elements (EREs) [2]. Liganded ER undergoes conformational changes which facilitate cofactor recruitment [3], and forms multi-subunit protein complexes [4], [5], [6]. Many of these co-regulators are enzymes that alter chromatin structure or control sequential transcriptional reactions [1], [7], which include ATP-dependent chromatin remodeling complexes (SWI/SNF) [8]. However, the molecular mechanisms of how co-regulators are recruited to the specific genes and how the integrated signals are transmitted to chromatin are not fully understood.

Genome-wide analyses of chromatin modifications have revealed a complex landscape of modified histones at transcription start sites (TSSs), distal regulatory elements and conserved sequences. In general, methylated H3K4 and H3K36 are associated with active transcription, whereas methylated H3K9, H3K27 and H4K20 are associated with gene silencing. H3K4me3 and H3K27me3 signals peak near TSSs. The relative presence of H3K4me3 and H3K27me3 has been shown to affect promoter activity [9], [10],[11],[12],[13]. H3K9me3 is generally linked to gene silencing since H3K9me3 is enriched in heterochromatin and inactive genes [13], [14], [15].

Recent discoveries of histone demethylases have advanced our understanding of transcriptional regulation [16], [17], [18], [19]. Histone demethylases JHDM2A (also known as JMJD1A or KDM3A; new nomenclature [20]), LSD1/KDM1 and JMJD2C/KDM4C act as co-activators in androgen receptor (AR) signaling [17], [21], [22], [23]. In ER signaling, LSD1 and JMJD1A/KDM2A are required for the induction of ER target genes upon E2 stimulation. Both LSD1 and JMJD1A can demethylate H3K9me2 in the regulatory region of ER target genes, thereby releasing the transcriptional block [21]. However, how histone demethylases function in estrogen-responsive cells and/or tissues *in vivo* is still unclear.

Abnormalities in the methylation of histones by histone methyltransferases have been implicated in various cancers [24]. Thus, it is possible that dysregulation of LSD1 or JmjC-domain-containing histone demethylases could contribute to tumorigenesis. Here we characterize JMJD2B as a newly-appointed co-regulator of ERα signaling in breast cancer growth and mammary gland development.

## Materials and Methods

### Ethics Statement

All experimental procedures were approved by the local Ethical Committee for Animal Experimentation of Ontario Cancer Institute (OCI), University Health Network (AUP#2031). The research projects that are approved by the local Ethical Committee for Animal Experimentation of OCI are operated in accordance with applicable Federal, Provincial, Municipal and Institutional regulations, the Policies and Guidelines of the Canadian Council on Animal Care and the Province of Ontario's Animals for Research Act. The animals are housed – accordance with applicable Federal, Provincial, Municipal and Institutional regulations, the Policies and Guidelines of the Canadian Council on Animal Care and the Province of Ontario's Animals for Research Act in the OCI Animal Facilities of the university. The Institute is committed to the highest ethical standards of care for animals used for the purpose of continued progress in the field of human medicine.

### Antibodies and primers

All antibodies and primers used are described in Procedure S1.

### Database search for JMJD2B expression in breast cancers

The ONCOMINE database and gene microarray analysis tool, a repository for published complementary DNA microarray data (http://www.oncomine.org) [25], [26], was searched to retrieve information on *JMJD2B* mRNA expression in human breast cancers. Statistical analysis of the differences in *JMJD2B* expression between ER-positive and ER-negative breast cancers was performed using ONCOMINE algorithms that allow for multiple comparisons among different studies.

### Cell lines and culture conditions

MCF-7 and T-47D human breast cancer cells were obtained from American Type Culture Collection (Manassas, VA). MCF-7 cells were cultured in Eagle's Minimum Essential Medium (ATCC #30-2003) supplemented with antibiotics, 10% fetal bovine serum (FBS) and 0.01 mg/mL bovine insulin. T-47D cells were cultured in RPMI-1640 supplemented with antibiotics, 10% FBS and 0.2 units/mL bovine insulin. For steroid-free medium, phenol red-free DMEM (Gibco) and charcoal/dextran-treated FBS (HyClone, SH30068) were used.

### Real-time RT-PCR

RNA was extracted from cells using an RNeasy Mini kit (Qiagen). Total cellular RNA was converted into cDNA by reverse transcription (Superscript III; Invitrogen Life Technologies) using random primers. PCR amplification was performed using Power SYBR Green qPCR SuperMix-UDG with ROX (Invitrogen Life Technologies) through 40 cycles of 95°C for 15 s and 60°C for 60 s using an Applied Biosystems PRISM 7900 Sequence Detection System.

### RNA interference

siRNAs for JMJD2A and JMJD2B and non-targeted control siRNA were purchased from Dharmacon. Validations of the siRNAs are in Figures S1A, S1B, S1C, S1E and S1F. For JMJD2B shRNA, the pLKO.1 puro (Addgene plasmid #8453) [27] lentiviral vector and control scrambled shRNA (Addgene plasmid #1864) [28] were used. Lentivirus was prepared by transfecting 293T cells with the knockdown vectors, pMD2.G (Addgene plasmid #12259) and psPAX2 (Addgene plasmid #12260). Target sequences are in Procedure S1.

### Soft agar assay

ZR-75-1 cells, MCF-7 cells, or derivative cell lines were cultured in 12-well plates containing a bottom agar layer consisting of culture medium plus 0.7% agar and 2 ng/mL puromycin. The middle layer contained 10^5^ cells in culture medium plus 0.35% agar and 1 ng/mL puromycin. Medium alone was added as the top layer to prevent desiccation of the agarose. Colonies were allowed to form for 14 days prior to visualization by crystal violet staining.

### Mouse Xenograft Breast Cancer Models

Slow-release estradiol pellets were implanted subcutaneously into NIH-III mice (Crl:NIH-Lyst^bg^Foxn1^nu^Btk^xid^, 7-week old, Charles River Laboratories) three days before tumor transplantation. 3×10^6^ ZR-75-1 cells grown in cell culture were suspended in 50 µL medium, mixed with 50 µL Matrigel, and injected subcutaneously into hind flanks. Tumors derived from injected cells were harvested two weeks after transplantation.

### BrdU/7-AAD staining

For cell cycle analysis using BrdU and 7-amino-actinomycin D (7-AAD), cells were pulsed with 10 µM BrdU for 1 hr. The FITC BrdU flow kit (BD Biosciences) was used to detect BrdU. Fluoresence-activated cell sorting (FACS) analysis was performed on a FACSCalibur (Becton Dickinson), and data were analyzed with Cellquest or FlowJoe software.

### Immunoprecipitation assay

Cells were washed with PBS, scraped off, and collected by centrifugation. The cells were suspended in hypotonic buffer (10 mM Tris-HCl (pH 7.4), 10 mM NaCl, 3 mM MgCl_2_, 0.5% NP-40) in the presence of protease inhibitors (Complete(R), Roche). After washing with PBS, the pellet was resuspended in 1 mL of NP-40 IP lysis buffer (50 mM TrisHCl (pH 7.6), 150 mM NaCl, 1% NP-40, 1 mM EDTA) containing protease inhibitors. The lysate was sonicated at 5% output for 20 sec. After centrifugation, the supernatant was collected and precleared by incubating with protein A magnetic beads (Dynabeads®, Invitrogen) at 4°C for 1 hr. Fresh protein A beads were washed with PBS-T (PBS+0.05% Tween) three times, resuspended in 100 µL PBS-T, and incubated with 5 µg of indicated antibodies at 4°C for 1 hr. The antibody-coated beads were then washed with PBS-T three times and incubated with 800 µL of the lysate at 4°C over night. The rest of the samples were kept for INPUT. After extensive wash, the beads were suspended in 80 µL of 2× sample buffer and boiled at 95°C for 5 min to elute associated proteins. To elute JMJD2C, the beads were incubated with 40 µL of 8 M Urea for 15 min at 37°C, and then boiled with 40 µL of 4× sample buffer.

### ChIP assay

Cells were crosslinked in 1% formaldehyde for 10 min at room temperature. Cells were lysed and sonicated to obtain fragmented chromatin samples, which were immunoprecipitated using specific antibodies coupled to protein A Dynabeads (Invitrogen). The protein-DNA complexes were eluted from the beads and incubated at 65°C overnight to reverse protein-DNA crosslinks. Precipitated DNA was analyzed by real-time PCR. Refer to Procedure S1 for primer information. The full version of ChIP assay procedure is in Procedure S1.

### ERα binding site analysis

Putative ER binding sites were identified by analyzing the bed files (http://research4.dfci.harvard.edu/brownlab/datasets; http://www.ncbi.nlm.nih.gov/pubmed/19005469) using UCSC genome browser. ERα responsive elements (EREs) were determined by Mapper (http://bio.chip.org/mapper).

### Gene targeting in mice

A *Jmjd2b*-targeting vector was constructed in which the *neo* cassette was flanked by *frt* sequences, and *Jmjd2b* exon 5 was flanked by *loxP* sequences (Figure S6A). Cre-mediated removal of exon 5 results in a frameshift and translation termination, and removal of H189 and E191 residues essential for demethylase activity. Homologous recombination in ES cells and germ line transmission were confirmed by Southern blot analysis (Figures S6B abd S6C). The full version of the gene targeting strategy is Procedures S1.

### Isolation of mammary epithelial cells

Mammary fat pads were minced into paste, digested in collagenase/hyaluronidase solution (StemCell Technologies, 07912), and dissociated with dispase (StemCell Technologies, 07913). Samples were washed with Hanks' Balanced Salt Solution supplemented with 2% FBS and 2 mmol/L EDTA (HFE) and centrifuged at 300×*g* for 5 min. The pellet was washed with HFE and then passed through a 40 µm cell strainer to obtain a single-cell suspension. CD45^+^Ter119^+^ and CD31^+^ cells were removed using the EasySep biotin selection kit (StemCell Technologies) to obtain Lin^−^ cells.

## Results

**Figure 1:**
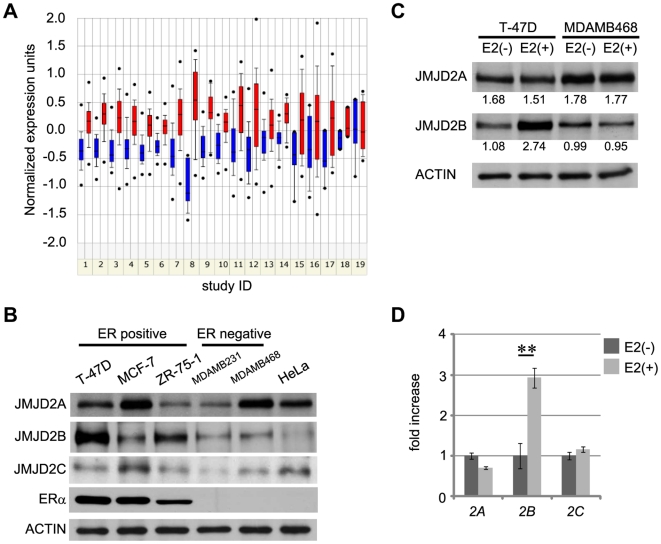
Elevated expression of JMJD2B in ER-positive breast cancers. (A) *JMJD2B* mRNA expression in clinical breast cancer samples from 19 studies registered in the ONCOMINE database. Blue bars, *JMJD2B* mRNA in ER-negative samples; red bars, *JMJD2B* mRNA in ER-positive samples. Data were analyzed using ONCOMINE algorithms. mRNA expression levels are represented by normalized units. The line within each colored box represents the median value for each group, and the upper and lower edges of each box indicate the 75th and 25th percentiles, respectively. Study details and statistical values are described in Table S1. (B) JMJD2B protein levels are higher in ER-positive breast cancer cell lines. Extracts of ER-positive and ER-negative breast cancer cell lines were analyzed by western blot to detect JMJD2 family members. Actin, loading control. (C) JMJD2B protein expression is induced by E2 stimulation. T-47D cells (ER-positive) and MDA-MB468 cells (ER-negative) were cultured in steroid-depleted serum for 4 days and stimulated with or without E2 for 24 hr. Cell extracts were analyzed by western blot. Relative intensity of the JMJD2A and JMJD2B bands are shown. The values are normalized to the bands of corresponding actin. (D) E2 stimulates *JMJD2B* expression. T-47D cells were cultured in steroid-depleted serum for 4 days followed by E2 stimulation for 6 hr. *JMJD2A*, *JMJD2B* and *JMJD2C* transcript levels are shown relative to levels in untreated controls (set to a value of 1). Expression levels were normalized to *ACTB* expression levels. Data represent mean ± s.d. of triplicates. ** p<0.01. Full-length blots are presented in Figure S7.

As a first step towards identifying signature differences between ER-positive and ER-negative breast cancers, we searched the ONCOMINE database [25]–[26] and found 19 suitable studies (Table S1). In most of these studies, expression of *JMJD2B* mRNA was higher in ER-positive cancers than in ER-negative cancers (Figure 1A; refer to Table S1 for p-values). We examined protein levels of JMJD2 family members in breast cancer cell lines. JMJD2B expression was generally higher in ER-positive than in ER-negative lines (Figure 1B). Furthermore, JMJD2B protein levels increased in response to E2 treatment in the ER-positive breast cancer cell line T-47D, but not in the ER-negative breast cancer cell line MDA-MB468 (Figure 1C). Real-time RT-PCR analysis confirmed that *JMJD2B* mRNA, but not *JMJD2A* or *JMJD2C* mRNA, was induced upon E2 treatment (Figure 1D).

**Figure 2:**
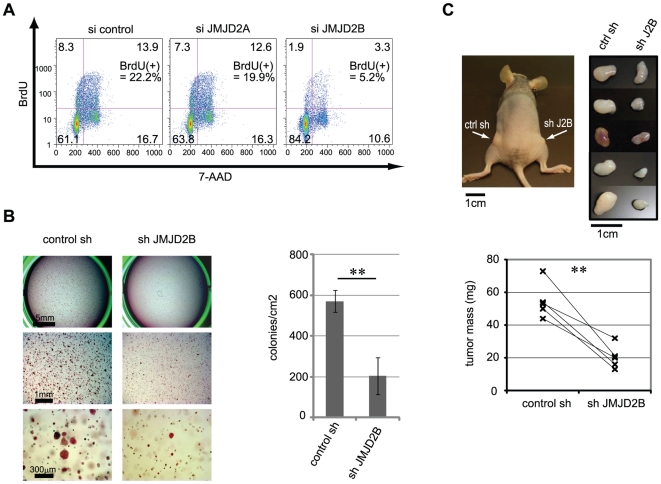
JMJD2B positively regulates the proliferation of ER-positive breast cancer cells. (A) JMJD2B knockdown impairs proliferation. T-47D cells were transfected with either control siRNA or siRNA against JMJD2A or JMJD2B, cultured for 72 hr, pulsed for 1 hr with BrdU, stained with APC-conjugated anti-BrdU antibody and 7-AAD, and analyzed by flow cytometry. Results are representative of four independent trials. (B) JMJD2B knockdown impairs colony formation. Single cell suspensions of ZR-75-1 cells expressing control shRNA or shRNA against JMJD2B were seeded in soft agar. After 14 days, colonies were stained with crystal violet and microscopic fields were photographed (left). The number of colonies/cm^2^ was determined (right). Data represent the colony density of three wells (mean ± s.d). **p<0.01. Representative data of three independent trials. See also Figure S2. (C) JMJD2B knockdown impairs tumor formation in xenograft model. Upper left: tumors (indicated by arrows) in a representative NIH-III mouse injected with control ZR-75-1 cells on the left flank and JMJD2B-depleted ZR-75-1 cells on the right flank. Upper right: dissected tumors from nude mice. Lower: mass of tumors is shown. **p<0.01.

To understand the role of JMJD2B in regulating cancer cell growth, siRNA was used to knock down JMJD2B expression in T-47D cells (Figure S1A). At 72 hr post-siRNA transfection, we analyzed proliferation by BrdU/7-AAD staining and flow cytometry, and found that BrdU incorporation was substantially reduced in JMJD2B-depleted cells but not in control cells or JMJD2A-depleted cells (Figure 2A). To investigate the effects of prolonged JMJD2B depletion, we generated ZR-75-1 cells in which JMJD2B expression was knocked down using short hairpin RNA (shRNA; Figure S1D), and assessed the ability of these cells to form colonies in soft agar. JMJD2B-depleted ZR-75-1 cells formed fewer and smaller colonies relative to control (Figure 2B). Reduced colony formation was also observed for MCF-7 cells expressing JMJD2B-targeted shRNA (Figure S2).

We performed xenograft experiments in which NIH-III mice were subcutaneously implanted with slow-release estradiol pellets and injected with ZR-75-1 cells expressing control shRNA and shRNA against JMJD2B. The two cell lines were injected into opposing flanks of the same mouse. JMJD2B-depeleted cells gave rise to significantly smaller tumors than cells expressing control shRNA (Figure 2C). These results demonstrate that JMJD2B positively regulates the proliferation of ER-positive breast cancer cell lines.

**Figure 3:**
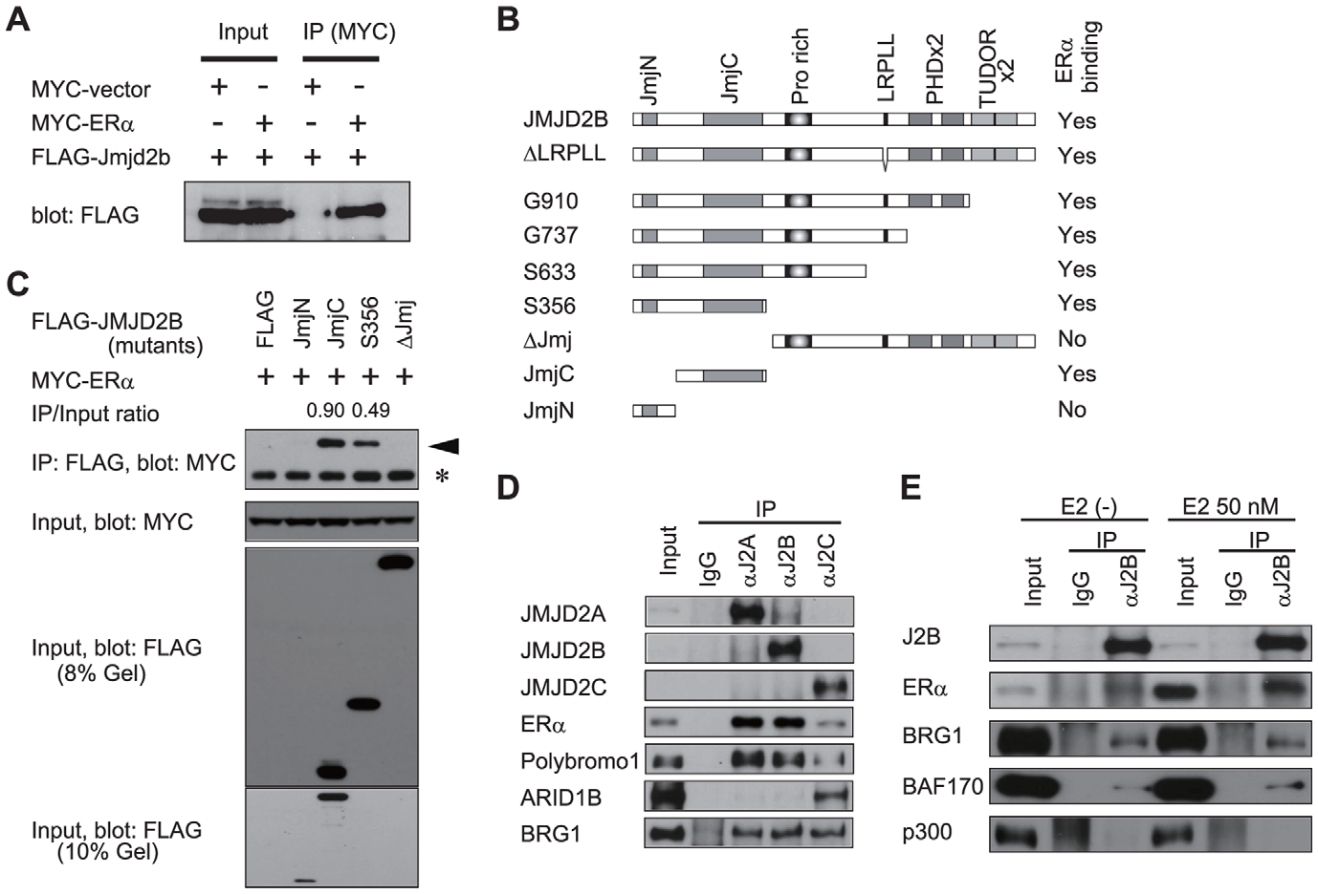
JMJD2B interacts with ERα and SWI/SNF-B complex. (A) JMJD2B co-immunoprecipitates with ERα. 293T cells were co-transfected with expression plasmids for FLAG-JMJD2B and either MYC-ERα or empty vector. Cell lysate and α-MYC immunoprecipitates were analyzed by western blot with α-FLAG antibody. (B) Structure of JMJD2B deletion mutants. Pro rich, proline rich region. LRPLL, LRPLL motif. PHDx2, double PHD domain. TUDORx2, double TUDOR domain. Western blot analysis of mutants is shown in Figure S3A. (C) The JmjC domain is sufficient for co-immunoprecipitation with ERα. 293T cells were co-transfected with MYC-ERα and individual JMJD2B deletion mutant expression vectors (refer to B). Cell lysates and α-FLAG immunoprecipitates were analyzed by western blot with antibodies to the indicated proteins. Relative intensity of the bands of the mutants is shown, normalized to the bands of corresponding input. Arrowheads, ERα bands; *, α-FLAG antibody. (D) Association of JMJD2 proteins with ERα, SWI/SNF complex, or SWI/SNF-B complex. (E) Association of JMJD2B with ERα or SWI/SNF-B complex in the absence or presence of estrogen simulation. (D and E) Nuclear lysates of T-47D cells were immunoprecipitated with control IgG or antibodies to the indicated proteins. Input lysate and immunoprecipitated samples were immunoblotted using antibodies to the indicated proteins. See also Figure S3. Full-length blots are in Figure S7.

JMJD2C associates with androgen receptor and is required for its transcriptional activity [29]. Furthermore, knockdown of JMJD2C in prostate cancer cell lines impairs the response to androgen receptor ligand [29]. We, therefore, speculated that JMJD2B may be required for ER transcriptional activity. We co-transfected 293T cells with MYC-tagged ERα and FLAG-tagged JMJD2B, and observed that JMJD2B co-immunoprecipitated with ERα (Figure 3A). We then generated a series of FLAG-tagged JMJD2B mutant proteins bearing carboxy-terminal deletions (Figure 3B) and tested them for co-immunoprecipitation with ERα. The JMJD2B protein contains N-terminal JmjN and JmjC domains, a central Pro-rich domain, and C-terminal double PHD and double Tudor domains (Figure 3B). We found that only the JmjC domain was required for the interaction with ERα (Figures 3C and S3D). We then tested whether ERα interacts with a catalytically inactive JMJD2B mutant (ΔFeJMJD2B; Figure S3F) which contains point mutations (H189Y and E191A) in the iron-binding region [30], [31], [32], [33]. The data suggest that catalytic activity of JMJD2B does not affect the interaction with ERα (Figure S3A). We found that the benzonase treatment did not influence the interaction between ERα and JMJD2B (Figures S3B and S3C). These data demonstrate that the ERα-JMJD2B interaction is DNA independent.

Given that the JmjC domain of JMJD2B mediates the interaction with ERα and that the JmjC domain is conserved among JMJD2 proteins, it is possible that JMJD2A and JMJD2C also interact with ERα. To address this issue, nuclear proteins were extracted from T-47D cells and co-immunoprecipitation experiments were performed with antibodies specific to endogenous proteins. Antibodies to JMJD2A or JMJD2B immunoprecipitated a substantial amount of ERα. In contrast, complexes between JMJD2C and ERα appeared to be less abundant (Figure 3D). We also performed co-immunoprecipitation assay for the extracts treated with benzoase.

ERα recruits the SWI/SNF chromatin remodeling complex (BAF complex) to target genes upon estrogen stimulation. We found that JMJD2A, JMJD2B, and JMJD2C interacted with one of the core subunits of SWI/SNF complex, BRG1 ATPase (Figure 3D). JMJD2A and JMJD2B also interacted with Polybromo1 but not ARID1B, whereas JMJD2C interacted with ARID1B and, to a lesser extent, Polybromo1 (Figure 3D). Because Polybromo1 is specific to the SWI/SNF-B complex (P-BAF complex) and ARID1B is specific to the SWI/SNF complex, these results suggest that JMJD2A and JMJD2B associate specifically with the SWI/SNF-B complex whereas JMJD2C associates preferentially with the SWI/SNF complex. We further examined whether formation of these JMJD2B-containing complexes is estrogen dependent. Estrogen treatment resulted in nuclear accumulation of ERα and an increased association between ERα and JMJD2B (Figures 3E and S3E). In contrast, estrogen stimulation did not affect the interaction between JMJD2B and subunits of the SWI/SNF complex (Figures 3E and S3E), suggesting that JMJD2B can interact with the SWI/SNF-B complex in the absence of estrogen stimulation. We could not detect an interaction between JMJD2B and p300 (Figure 3E) which is supposedly recruited to ERα with kinetics distinct to those of the SWI/SNF complex [7].

**Figure 4:**
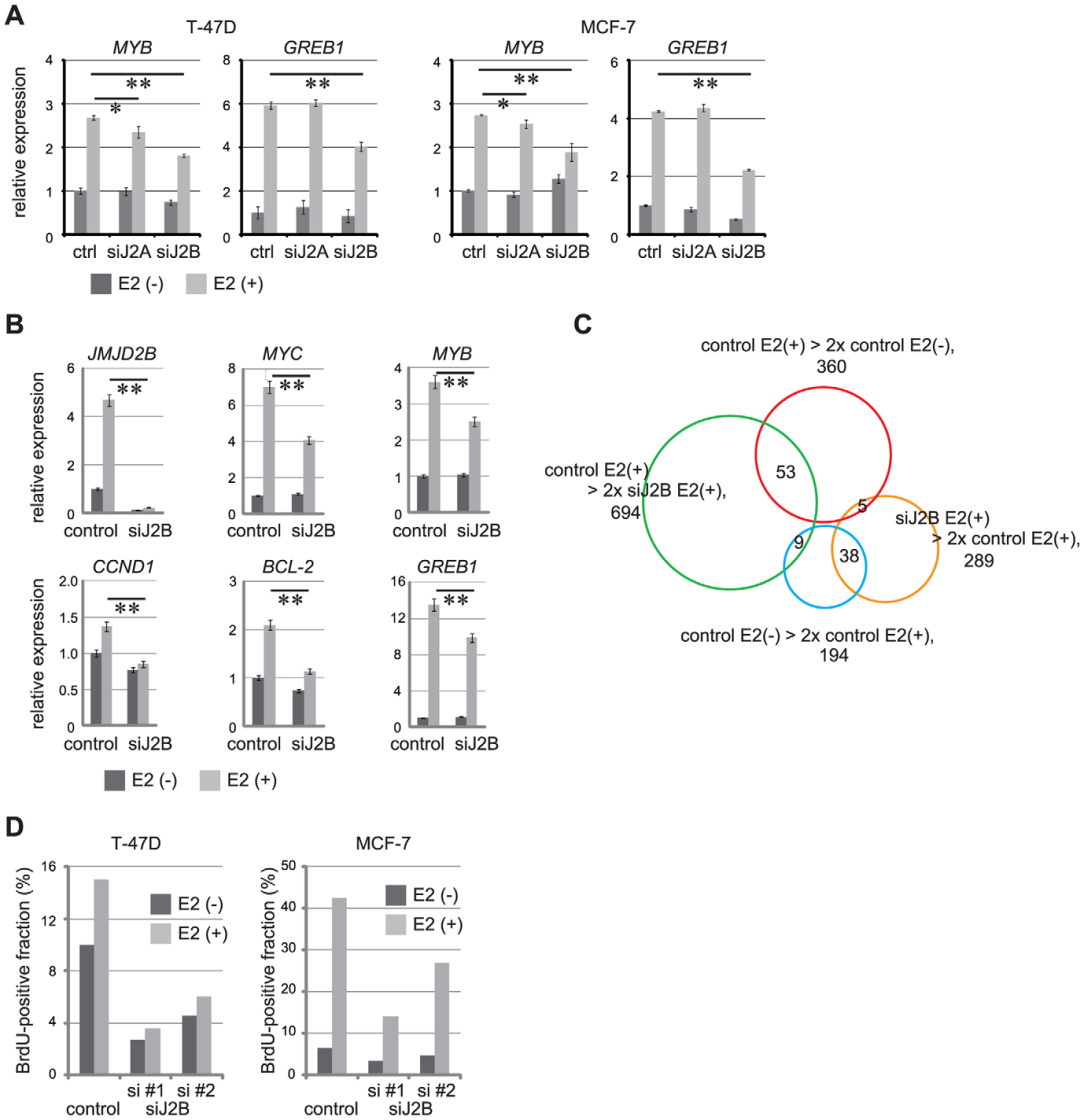
JMJD2B mediates induction of ER target genes and estrogen-dependent proliferation of breast cancer cells. (A) Effect of JMJD2A or JMJD2B knockdown on induction of ER target genes. T-47D cells and MCF-7 cells were transfected with either control siRNA, JMJD2A siRNA or JMJD2B siRNA, cultured in steroid-free medium for 72 hr, and stimulated with or without E2 for 4 hr. (B) JMJD2B knockdown impairs ER target gene induction. T-47D cells were transfected with either control siRNA or JMJD2B siRNA, cultured in steroid-free medium for 72 hr, and stimulated with or without E2 for 4 hr. (A and B) mRNA levels of *JMJD2B* or the indicated ER target genes were measured by real-time RT-PCR. Results represent mRNA levels normalized to the level of *ACTB* mRNA (mean ± s.d. of triplicates). **p<0.01. (C) Venn diagram representation of the differentially expressed genes. The area of each circle reflects the number of genes contained in the respective categories. The heat map is in Figure S4C. The lists of the genes are in Tables S2, S3, S4, S5. (D) JMJD2B knockdown impairs cellular response to estrogen. T-47D cells or MCF-7 cells transfected with control siRNA or JMJD2B siRNA were cultured in steroid-free medium for 48 hr, stimulated for 24 hr with E2, labeled for 1 hr with BrdU, and stained with anti-BrdU antibody and 7-AAD. The fraction of BrdU-positive cells was determined by flow cytometry. A representative result from three independent experiments.

We next assessed whether JMJD2B is required for activation of ER target genes. Induction of *GREB1* in response to E2 was significantly reduced in JMJD2B-depleted T-47D cells and JMJD2B-depleted MCF-7 cells but not in JMJD2A-depleted cells (Figure 4A). *MYB* oncogene is an ER target gene and required for ER-induced cell proliferation [34]. The induction of *MYB* in T-47D cells and MCF-7 cells was impaired by JMJD2B knockdown, whereas JMJD2A knockdown impaired *MYB* induction to a modest degree (Figure 4A). These results are consistent with the observation that knockdown of JMJD2A had little effect on the proliferation of T-47D cells (Figure 2A). Collectively, these results indicate that JMJD2B is required for the full extent of ERα transcriptional activity and that the JMJD2A has only a limited role in the transcriptional activity of ERα despite a comparable affinity for ER and SWI/SNF-B complexes. Therefore, we focused our attention on the function of JMJD2B. Induction of other ER target genes including *MYC*, *CCND1*, and *BCL-2* was also reduced in JMJD2B-depleted T-47D cells (Figure 4B) and JMJD2B-depeleted MCF-7 cells (Figure S4A).

To further assess the effect of JMJD2B depletion, we performed genome-wide gene expression analysis using the Affymetrix Human Gene 1.0 ST array. RNA was extracted from steroid-depleted control MCF-7 cells (control E2(−)), control MCF-7 cells treated with E2 (control E2(+)), steroid-depleted JMJD2B-depleted MCF-7 cells (siJ2B E2(−)), and JMJD2B-depleted MCF-7 cells treated with E2 (siJ2B E2(+)). After normalizing the data, we identified genes that exhibited a log-fold change of greater than 2 between control E2(−) and control E2(+) cells, or between control E2(+) and siJ2B E2(+) cells. (The false discovery rate (FDR) was set at <0.05.) In total, we identified 1432 differentially expressed genes (Figures 4C and S4C; Table S2, S3, S4). 878 genes were up-regulated or down-regulated by JMJD2B knockdown, but not affected by E2 stimulation. These genes represent candidate JMJD2B target genes that are independent of ER signaling. 360 genes were induced by E2, whereas 194 genes were down-regulated by E2. Among these 360 genes, 53 genes (14.7%) were down-regulated in siJ2B E2(+) relative to control E2(+) cells (Figure 4C), indicating that a subset of E2-induced genes were dependent on JMJD2B for full induction. Again we observed impaired induction of *MYB*, *GREB1*, and *BCL2* in JMJD2B-depleted cells (Figure S4B), although the changes in transcript levels did not pass the criteria of <0.05 FDR and >2 log-fold difference.

To determine whether JMJD2B is required for cellular responses to estrogen, we stimulated JMJD2B-depleted T-47D and MCF-7 cells with E2 and measured BrdU incorporation at 72 hr post-transfection. E2 stimulated the proliferation of control cells but had a limited effect on the proliferation of JMJD2B-depleted cells (Figures 4D and S4D), indicating that JMJD2B is required for the proliferative response to estrogen. Since the induction of *MYB*, *MYC*, and *CCND1* genes were reduced in JMJD2B-depleted cells upon E2 treatment, impairment of G1/S transition appears to contribute the defective proliferation. It has been recently reported that JMJD2B-depletion also influences genes required for G2/M transition [35].

**Figure 5:**
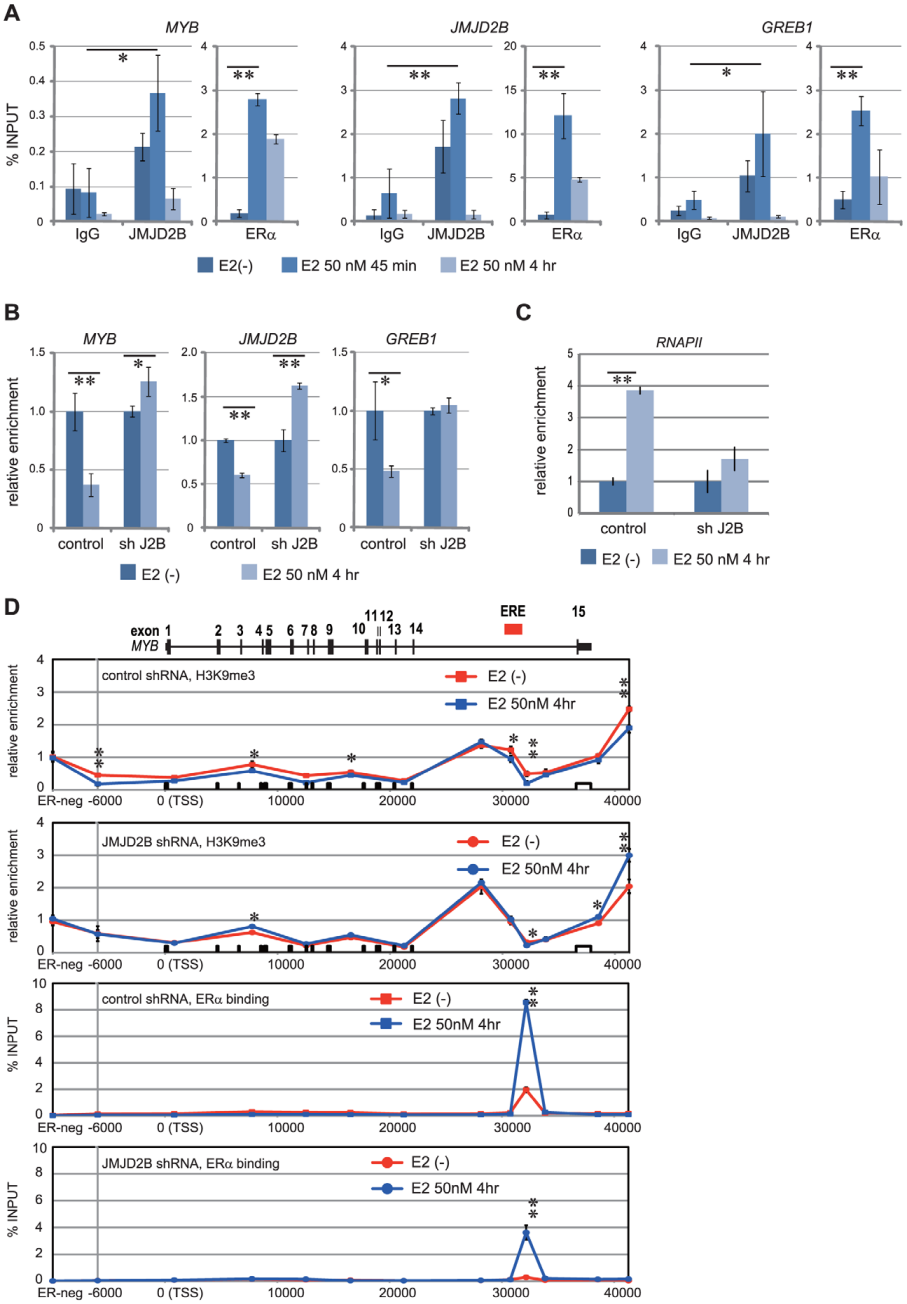
JMJD2B is recruited to the ER binding site; H3K9me3 is demethylated at the ER binding site. (A) Chromatin immunoprecipitation (ChIP) assay of ERα and JMJD2B at indicated gene loci. T-47D cells were steroid-depleted for 96 hr and treated with E2 for 45 min or 4 hr. mRNA levels of *JMJD2B* and the indicated ER target genes in the corresponding samples are in Figure S5B. (B) ChIP assay of H3K9me3 at indicated gene loci. T-47D cells expressing control shRNA or shRNA against JMJD2B were steroid-depleted for 96 hr and treated with E2 for 4 hr. Results were normalized to input values, and expressed relative to untreated controls (set at 1). (C) ChIP assay of RNAPII in *MYB* gene. T-47D cells expressing control shRNA or JMJD2B shRNA were treated as in (B). Results were normalized to input values, and expressed relative to untreated controls (set at 1). (D) Presence of ERα and H3K9me3 within the *MYB* locus in T-47D cells. A region previously shown to be devoid of ERα was chosen as a negative control site (ER-neg) [36]. Cells were treated as in (B). H3K9me3 ChIP signals were normalized to input chromatin and expressed relative to ChIP signals of the negative control site (set at 1). X-axis represents distance from TSS. (A–C) ChIP samples were quantified by real-time PCR. Data represent mean ± s.d. of triplicates. Representative data from three independent trials. **p<0.01; *p<0.05. ER binding sites assessed in this study are shown in Figure S5.

We next analyzed the interaction of ERα with target gene loci by performing chromatin immunoprecipitation (ChIP). We selected candidate ER binding sites according to the results of a published genome-wide analysis [36] and confirmed the binding of ERα to ER target genes including *MYB*, *JMJD2B*, and *GREB1* (Figure 5A). Recently, it has been reported that most estrogen receptor binding sites are distal to transcription start sites (TSSs). Indeed, ER binds to *MYB* and *GREB1* loci more than 20 kb downstream of their respective TSSs (Figure S5A). The interaction of ERα with these sites increased 45 min after E2 stimulation and then declined 4 hr later (Figure 5A). We also observed marked increase of JMJD2B binding to these sites following E2 stimulation (Figure 5A). Notably, ERα and JMJD2B were both detected at the ERα binding site in the *JMJD2B* locus, suggesting that JMJD2B regulates itself at the transcriptional level in concert with ERα, which is consistent with the previous genome wide analysis of ERα binding site [37].

We next addressed the demethylation of H3K9me3 at ER binding sites. We observed decreased H3K9me3 at ER binding sites following E2 stimulation in control but not in JMJD2B-depleted T-47D cells (Figure 5B), suggesting that JMJD2B regulates the demethylation of H3K9me3 at ER binding sites. H3 enrichment at *MYB* ERE appears to decrease over the time after E2 addition but H3 displacement during the time window we have analyzed is not statistically significant whereas H3K9me3/H3 ratio at *MYB* ERE decreases to approximately one-third in the presence of E2 (Figure S5C). We also examined RNA polymerase II (RNAPII) levels at upstream of the ER binding site in MYB gene. The RNAPII levels were markedly increased in control T47D cells in response to E2 treatment. Conversely, the induction of RNAPII levels was significantly impaired in JMJD2B-depleted cells (Figure 5C). These findings further support a role of JMJB2B in ER regulated transcription.

To further study the involvement of JMJD2B in the demethylation of H3K9me3 at an ER target gene [34], we performed ChIP on 12 regions within *MYB* locus. Loading of ERα occurred almost exclusively at the region identified above (Figure 5D). In control T-47D cells, H3K9me3 levels were reduced following E2 treatment at several regions of the *MYB* locus, including the ER binding site. In contrast, chromatin-bound H3K9me3 was not reduced in JMJD2B-depleted cells (Figure 5D). We also performed ChIP for ERα. We found that JMJD2B depletion reduced ERα enrichment (Figure 5D), suggesting that JMJD2B depletion reduces recruitment of ERα or stability of ERα complex.

Taken together, in response to E2 stimulation, JMJD2B is recruited to the ER binding site and demethylates H3K9me3 in the surrounding region, thus facilitating gene induction. In some cases, levels of chromatin-bound H3K9me3 were higher in JMJD2B-depleted cells (Figure 5B and 5D). Histone methyltransferases has been proposed to associate with ERα to establish a basal repressive state at ER targets [21]. It is possible that the increase in H3K9me3 levels was caused by the loss of the competing demethylase activity of JMJD2B.

**Figure 6:**
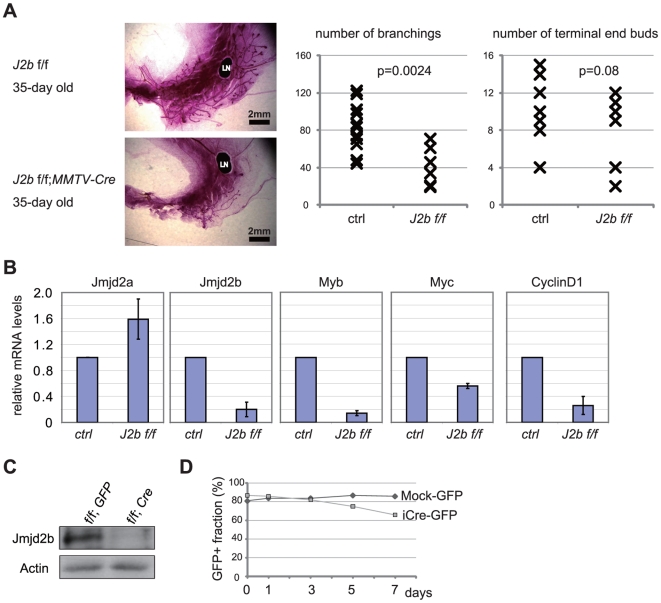
Conditional deletion of *Jmjd2b* in mammary epithelium results in defective mammary gland development. (A) Defective development of *Jmjd2b*-deficient mammary gland. Carmine-stained whole-mount preparations of mammary glands from 5-week old *Jmjd2b* ^flox/flox^;*MMTV-Cre* and *Jmjd2b* ^flox/flox^ mice (Ctrl∶control). Representative littermates are shown. LN, lymph node. The numbers of branching and terminal end buds within a fat pad were determined (*Jmjd2b* ^flox/flox^ n = 11; *MMTV-Cre*;*Jmjd2b* ^flox/flox^: n = 6). (B) Impaired gene expression in *Jmjd2b* ^flox/flox^; *MMTV-Cre* MECs. Lin^−^ MECs were isolated from *Jmjd2b* ^flox/flox^;*MMTV-Cre* and *Jmjd2b* ^flox/flox^ mice. mRNA levels of *Jmjd2b*, *Myb*, *CyclinD1* and *c-myc* were examined by real-time RT-PCR. Results represent mRNA levels normalized to the level of *Actb* mRNA (mean ± s.d. of five independent littermate pairs). (C) Western blot showing the absence of Jmjd2b protein in Cre-infected *Jmjd2b* ^flox/flox^ MECs. Full-length blots are in Figure S7 (D) Impaired proliferation of *Jmjd2b*-deficient MECs. Lin^−^ MECs were isolated from *Jmjd2b* ^flox/flox^ mice, infected with Mock-*GFP* or *Cre-IRES-GFP* (iCre-GFP) retrovirus, and cultured *in vitro*. Fractions of GFP-positive cells were determined at the indicated time points by FACS analysis. A representative result from three independent experiments is shown.

To determine whether JMJD2B is required for the proliferation of normal epithelial cells under physiological conditions, we generated mice carrying a conditional allele of *Jmjd2b* that can be removed in mammary epithelial cells (MECs) by expression of *Cre* recombinase under the control of the MMTV promoter (*Jmjd2b* ^flox/flox^;*MMTV-Cre* mice; Figure S6). We chose to flank *Jmjd2b* exon 5 with *loxP* sequences because this exon encodes a fragment containing H189 and E191 residues that are essential for iron-binding and demethylase activity [30], [31], [32], [33]. We analyzed whole-mount specimens of mammary fat pads and noticed that mammary gland development was delayed in *Jmjd2b* ^flox/flox^; *MMTV-Cre* mice (Figure 6A). Relative to control mice, *Jmjd2b* ^flox/flox^;*MTV-Cre* mice exhibited reduced branching in the mammary gland (Figure 6A), which is consistent with a previous report that ERα is required for ductal morphogenesis [38].

We next examined gene expression in isolated MECs. We isolated CD45-Ter119-CD31- (Lin−) MECs [39], [40], [41], and found that mRNA levels of *Myb*, *CyclinD1* and *c-myc* were significantly reduced in *Jmjd2b* ^flox/flox^;*MMTV-Cre* mice compared to *Jmjd2b* ^flox/flox^ mice (Figure 6B). These data further support that the defective mammary gland development is due to the impairment of the ER target genes induction. However, further study is required to clarify whether *Jmjd2b*-deficiency primarily leads to cell proliferation defect, apoptosis, or both.

To further assess impact of *Jmjd2b*-deficiency on proliferation of MECs, the isolated *Jmjd2b* ^flox/flox^ MECs were infected with either Mock-*GFP* (control) or *iCre-IRES-GFP* retrovirus, and then cultured *in vitro*. Western blotting confirmed that Jmjd2b protein was absent from cells infected with *Cre*-expressing virus (Figure 6C). FACS was used to monitor the fraction of GFP-positive cells. The proportion of GFP-positive control cells did not change over time, whereas that of *iCre*-infected, GFP-positive cells gradually decreased, suggesting a role for Jmjd2b in the proliferation of mammary epithelial cells (Figure 6D).

It is noteworthy that we have obtained viable *Jmjd2b−/−* mice without gross abnormalities (data not shown), indicating that Jmjd2b is not a general regulator of cell proliferation. These observations further support our findings that Jmjd2b has tissue and signal specific functions.

## Discussion

Intensive studies of how nuclear receptors transmit ligand binding signals have identified several distinct multiprotein complexes. These complexes are recruited to promoters and enhancers, and modulate higher-order chromatin and nucleosomal structures leading to activation or repression of target genes. Genome-wide analysis of ERα binding sites has been performed by numerous groups and potential ERE sites have been mapped in promoter, enhancer and intronic regions; however, how the distal regulatory EREs function and what are co-factors at these sites remain to be elucidated.

Here, we describe indispensable roles for an H3K9 histone demetylase, JMJD2B, in ER signaling. JMJD2B was required for the full induction of ER target genes that function in cell proliferation and survival pathways (Figure 4). Consequently, inactivation of JMJD2B severely impaired the growth of ER-positive breast cancer and development of normal breast tissues *in vivo* (Figures 2 and 6). These findings are consistent with a recent report that JMJD2B depletion causes decrease in cell proliferation and colony formation capacity of ER-positive breast cancer cells [35].

Further, our studies address the mechanisms by which JMJD2B acts as a co-regulator in ER signaling. We observed that once ligand-bound ERα translocated to the nucleus, JMJD2B was recruited to distal ER binding sites of target genes concomitant with a decrease in chromatin-bound H3K9me3 levels (Figure 5). In agreement with these findings, depletion of JMJD2B severely impaired the demethylation of H3K9me3 in ER-binding regions upon E2 treatment (Figure 5). In addition to ERα, JMJD2B also associated with members of a chromatin remodeling complex, SWI/SNF-B, which is required for transactivation by nuclear receptors [42]. We therefore propose a model in which JMJD2B at ER binding sites removes the repressive histone marks, which may also set up docking sites for enzymes and transcription factors that remodel chromatin or otherwise modify gene-control regions.

### Diversity among JMJD2 family proteins

Recent studies have found that JMJD2 family proteins can target methylated H3K9 and H3K36 with some target specificity [43], suggesting that JMJD2 members have both common and distinct biological functions. Biochemically, JMJD2A/KDM4A and JMJD2C/KDM4C can demethylate H3K9me3 and H3K36me3 [33], [43], [44], whereas JMJD2B/KDM4B is likely more specific for H3K9me3 *in vitro* [43] although overexpression of JMJD2B demethylates H3K36me3 in cells [31]. Functionally, JMJD2A regulates the balance of H3K9me3 and H3K36me3 required for maintaining genomic stability in germ cells [43]. JMJD2B has been shown to antagonize H3K9me3 at heterochromatin [31]. JMJD2C promotes AR signaling [29]. Our data identify a specific role for JMJD2B in ER signaling. Among the JMJD2 family members we tested, JMJD2A and 2B exhibited robust interactions with ER (Figure 3); however, in contrast to depletion of JMJD2B, depletion of JMJD2A caused only a marginal defect in ER target gene induction (Figure 4).

JMJD2A and JMJD2B, although expressed, were not found to be involved in AR signaling [29]. Our data strongly suggest that JMJD2C (SWI/SNF-A) and JMJD2B (SWI/SNF-B) associate with different remodeling complexes. Estrogen may also further enhance JMJD2B expression to drive the vicious cycle. These findings might explain the specificity of ligand-receptor signaling and the mutually exclusive pathological implications involving JMJD2B and JMJD2C. Further investigation will be required to elucidate the signal and target specificity of each JMJD2 family member.

### Roles of histone lysine methylation in ER signaling

Genome-wide ChIP analysis of ER binding regions [36], [37], [45] and ChIP-sequence studies of RNAPII recruitment [46], [47] have revealed that, in addition to ERα binding, modifications of local chromatin and epigenetic states, recruitment of specific co-factors and release of RNAPII pausing are critical for the transcriptional regulation of ER responsive genes.

Enrichment of H3K9me3 has been reported in coding regions of some active genes [48], [49]; however recent genome-wide investigations support the general repressive nature of the H3K9me3 mark [13], [15]. Increasing recent evidence indicates that genomic architecture is not colinear but is modular [50], and permissive and repressive chromatin regions exist within the body of gene. The intragenic permissive chromatin regions are flanked by the repressive mark, H3K9me3, and the maintenance of the intragenic chromatin boundary appear to functions as a checkpoint in elongation [51]. We observed enrichment of H3K9me3 near the distal ER binding site under steroid-depleted condition, and found that the methylation levels significantly decreased upon E2 stimulation in a JMJD2B dependent manner (Figures 5B and 5D). In addition, JMJD2B-depletion reduced RNAPII occupancy (Figure 5C). Thus, our data suggest that JMJD2B contributes the establishment of local epigenetic state and chromatin structure, which are required for proper induction of ER responsive genes.

We observed the binding of ERα and JMJD2B to ER binding regions in the absence of estrogen stimulation although the binding increased upon estrogen stimulation (Figure 5A). LSD1 and JMJD2C have been shown to present at promoter and enhancer regions of AR-dependent genes prior to ligand stimulation. However, ligand-receptor interaction is needed to induce AR recruitment and subsequent demethylation [22], [29]. In an analogous way, recruitment of liganded ER and other co-factors may be necessary for JMJD2B activity. Further genome-wide analysis will be required to clarify these issues.

### Biological significances of JMJD2B in human diseases

Recent studies have implicated H3K9 modifications in numerous biological phenomena including germ cell development, X chromosome inactivation, DNA damage repair and apoptosis [14]. Recent reports also link deregulated histone methylation to tumorigenesis [52], [53].

Interestingly, focal amplification of JMJD2B locus was identified in brain tumors [54]. An H3K9 histone methyltransferase, Suv39H1, has been shown to function as a tumor suppressor by maintaining H3K9 methylation levels [55], [56]. In addition, we show that JMJD2B enhances the transcriptional activation of oncogenes and anti-apoptotic genes. Dysregulation of JMJD2 thus may counteract the tumor suppressive function of Suv39H1.

In conclusion, our data indicate that JMJD2B regulates an epigenetic signature in ER binding regions distal to TSSs leading to the establishment of specific transcriptional programs and biological effects downstream of ER signaling. In addition, the recent report has identified that combination of expression levels of a hypoxic marker and JMJD2B predict patient survival [35].We thus believe that further characterization of JMJD2B and related signaling components may identify therapeutic targets and prognostic markers for human cancers.

## Supporting Information

Figure S1 **Validation of JMJD2B knockdown efficiency.** (A) T-47D cells were transfected with siRNA against JMJD2A (target sequences #1 and #2). (B) T-47D cells were transfected with siRNA against JMJD2B (target sequences #1, #2, and #6). (A and B) Total cell lysate was prepared 72 hr after transfection and subjected to western blotting. (C) T-47D cells were transfected with si JMJD2B #1 or #2. RNA was extracted 72 hr after transfection and subjected to quantitation by real-time RT-PCR. (D) MCF-7 cells were infected with a lentiviral vector harboring JMJD2B shRNA targeting sequence #1, #2 or #3. Total cell lysates were prepared 48 hr after infection and subjected to western blotting. (E) T-47D cells were transfected with siRNA against JMJD2B (target sequences #1, #2, and #6). Total cell lysate was prepared 72 hr after transfection and subjected to western blotting for JMJD2A. The introduction of #6 siRNA reduced JMJD2A protein levels. We therefore used #1 and #2 siRNA in this study. Positive control: protein samples from JMJD2A overexpressed cells. (F) T-47D cells were transfected with siRNA against JMJD2A (target sequences #1, #2, #3 and #4). Total cell lysate was prepared 72 hr after transfection and subjected to western blotting for JMJD2B.
(TIF)

Figure S2 **JMJD2B knockdown decreases colony formation of MCF-7 cells.** Single cell suspensions of MCF-7 cells expressing control shRNA or shRNA against JMJD2B (target sequence #2) were seeded in 0.35% soft agar. After 14 days, colonies were stained with crystal violet. Microscopic fields were photographed (left). The number of colonies/cm^2^ was determined (right). Data are the mean number of colonies formed in three wells ± s.d., p<0.01. A representative result from four independent trials.
(TIF)

Figure S3 **JMJD2B interacts with ERα and SWI/SNF-B complex.** (A) Catalytic activity of JMJD2B does not influence the interaction with ER. 293T cells were co-transfected with MYC-ERα and either empty vector, FLAG-JMJD2B or FLAG-ΔFeJMJD2B (a mutant with (H189Y and E191A) point mutations in the iron-binding region). α-MYC immunoprecipitates were analyzed by western blot with α-FLAG antibody. (B) Benzonase treatment does not affect the interaction of endogenous JMJD2B and ER. Nuclear lysates of T-47D cells were lyzed and sonicated in the presence or absence of 500 unit of Benzonase (Novagen) before immunoprecipitation with α-JMJD2B Ab. Input lysate and immunoprecipitated samples were immunoblotted using α-ERα antibody. Full-length blots are presented in Figure S7. (C) Sonication and benzonase treatment digest genomic DNA. Nuclear lysates were treated as in (B). 15% of treated and untreated samples applied for immunoprecipitation were loaded on 0.8% gel. The arrow indicates undigested genomic DNA. (D) The JmjN and JmjC domains are sufficient for co-immunoprecipitation with ERα. 293T cells were co-transfected with MYC-ERα and one of several JMJD2B deletion mutant expression vectors encoding the structures illustrated in Figure 3B. Cell lysates and α-FLAG immunoprecipitates were analyzed by western blot with antibodies against the indicated proteins. Relative intensity of the bands of the Flag-JMJD2B and mutants are shown, normalized to the bands of corresponding input. The values are presented wild type as 1. Arrowheads, ERα bands; arrows, wild type or deletion mutant JMJD2B proteins; asterisk, α-FLAG antibody. (E) Kinetics of Association between JMJD2B and ERα or SWI/SNF-B complex. Nuclear lysates were harvested at indicated time points after E2 stimulation and subjected to immunoprecipitation with control IgG or the antibodies against the indicated proteins. Input lysate and immunoprecipitated samples were then immunoblotted using antibodies against the indicated proteins. (F) JMJD2B reduces H3K9me3 levels. U2OS cells were transfected with FLAG-JMJD2B and FLAG-ΔFeJMJD2B and stained with anti-FLAG and anti-H3K9me3 antibodies followed by anti–mouse IgG (green) or anti–rabbit IgG (red). Nuclei were visualized using Hoechst 33258. Overlay: merge of FLAG and H3K9me3 staining. Data shown are representative of two independent preparations.
(TIF)

Figure S4 **JMJD2B mediates induction of ER target genes and estrogen-dependent proliferation of breast cancer cells.** (A) JMJD2B is required for the induction of ER target genes. MCF-7 cells were transfected with either control siRNA or JMJD2B siRNA (target sequence #1), cultured in steroid-free medium for 72 hr, and stimulated with or without E2 for 4 hr. mRNA levels of JMJD2B or the indicated ER target genes were measured by real-time RT-PCR. Results shown are mean mRNA level normalized to the amount of ACTB mRNA ± s.d. of triplicates. **, p<0.01. (B) Microarray data for representative ER target genes. Signal intensities on microarray for representative ER target genes are shown by their mean values (±SD). (C) Heat map representation of differentially expressed genes. One thousand four hundred and thirty-two differentially expressed genes (as calculated using a false discovery rate <0.05 and log-fold change >2) were sorted by hierarchical clustering. Each row represents a gene and each column represents a sample. Red indicates higher expression and blue lower expression. (D) JMJD2B knockdown impairs cellular response to estrogen. T-47D cells or MCF-7 cells transfected with control siRNA or JMJD2B siRNA were cultured in steroid-free medium for 48 hr, stimulated for 24 hr with E2, labeled for 1 hr with BrdU, and stained with anti-BrdU antibody and 7-AAD. The fraction of BrdU-positive cells was determined by flow cytometry. A representative result from three independent experiments.
(TIF)

Figure S5 **ER binding sites assessed in this study.** (A) The UCSC Genome browse map depicts the ER binding sites of *MYB* locus, *JMJD2B* locus, and *GREB1* locus. Depicted ER binding sites are identified in previously published studies. (B) Effect of JMJD2B knockdown on induction of ER target genes. mRNA levels of *JMJD2B* or the indicated ER target genes in the corresponding samples applied for the ChIP analysis are shown. (C) The change of H3 and H3K9me3/H3 ratio at *Myb* ERE.
(TIF)

Figure S6 **Conditional gene targeting of murine Jmjd2b locus.** (A) Introduction of *loxP* sites into *Jmjd2b*. A portion of the murine wild type *Jmjd2b* locus showing exon 5 (grey rectangle) and an 11.9 kb XbaI fragment are shown at the top. The targeting vector was designed to generate floxed exon5 (*loxP*; black triangles), to flank the *PGK-neo* cassette with *frt* sequences (grey triangles), and to introduce a new XbaI site (X). The targeted allele contains a diagnostic 7.7 kb XbaI fragment. The *neo* cassette was removed *in vivo* by FLPe recombinase as described in Materials and Methods. Cre-mediated recombination resulted in the deletion of *Jmjd2b* exon 5. The position of the 5′ flanking probe used for genotyping is indicated. (B) Southern blot analyses to identify *Jmjd2b* ^neo/+^ ES cells (lanes 2, 4, 5, and 6). Genomic DNA was digested with XbaI (upper panel) or EcoRI (lower panel) and hybridized with the probe indicated in (A). (C) Southern blot analyses to identify *Jmjd2b* ^neo/+^ mice (lanes 2 and 3), *Jmjd2b* ^flox/+^ mice (lanes 4 and 5), and *Jmjd2b* ^+/Δ^ mice (lanes 6 and 7). Genomic DNA was digested with XbaI (upper panel) or EcoRI (lower panel) and hybridized with the probe indicated in (A). The numbers in brackets indicate the ES clones from which the mice were derived.
(TIF)

Figure S7 **Full-length blots images.** Full-length blot images related to Figures 1, 3, S3 and 6 are shown. The cropped regions are indicated.
(TIF)

Table S1 **P value of difference in JMJD2B mRNA expression in ER-positive and ER-negative breast cancers in 19 ONCOMINE studies.**
(DOC)

Table S2 **Genes down regulated in JMJD2B depleted cells.**
(XLS)

Table S3 **Genes upregulated in JMJD2B depleted cells.**
(XLS)

Table S4 **Genes down regulated in E2 treated JMJD2B depleted cells.**
(XLS)

Table S5 **Genes upregulated in E2 treated JMJD2B depleted cells.**
(XLS)

Procedure S1 (DOC)
