# Correction: Histone Demethylase JMJD2B Functions as a Co-Factor of Estrogen Receptor in Breast Cancer Proliferation and Mammary Gland Development

**DOI:** 10.1371/journal.pone.0303780

**Published:** 2024-05-09

**Authors:** Masahito Kawazu, Kayoko Saso, Kit I. Tong, Tracy McQuire, Kouichiro Goto, Dong-Ok Son, Andrew Wakeham, Makoto Miyagishi, Tak W. Mak, Hitoshi Okada

The Fig 2A si Control panel [1] was erroneously duplicated to represent the Fig 2A si JMJD2A results. Additionally, upon review of the original data, a minor shift in the gating was identified due to a version change in FlowJo software, which has been resolved. The revised results are provided in the updated Fig 2 provided with this notice. Original data, including the original and revised gating, is provided in S1 File.

An expert member of the Editorial Board reviewed the updated figures and underlying data, and stated that the conclusions in [1] remain supported by the revised results.

The original underlying data to support all results in the article and Supporting Information files are available from the corresponding authors except for the data supporting the full-length blots in Figure S7, Southern blot analyses in Figure S6, and Figure 6A, 6D, which are no longer available.

The authors sincerely apologize for the errors in the published article.

**Fig 2:**
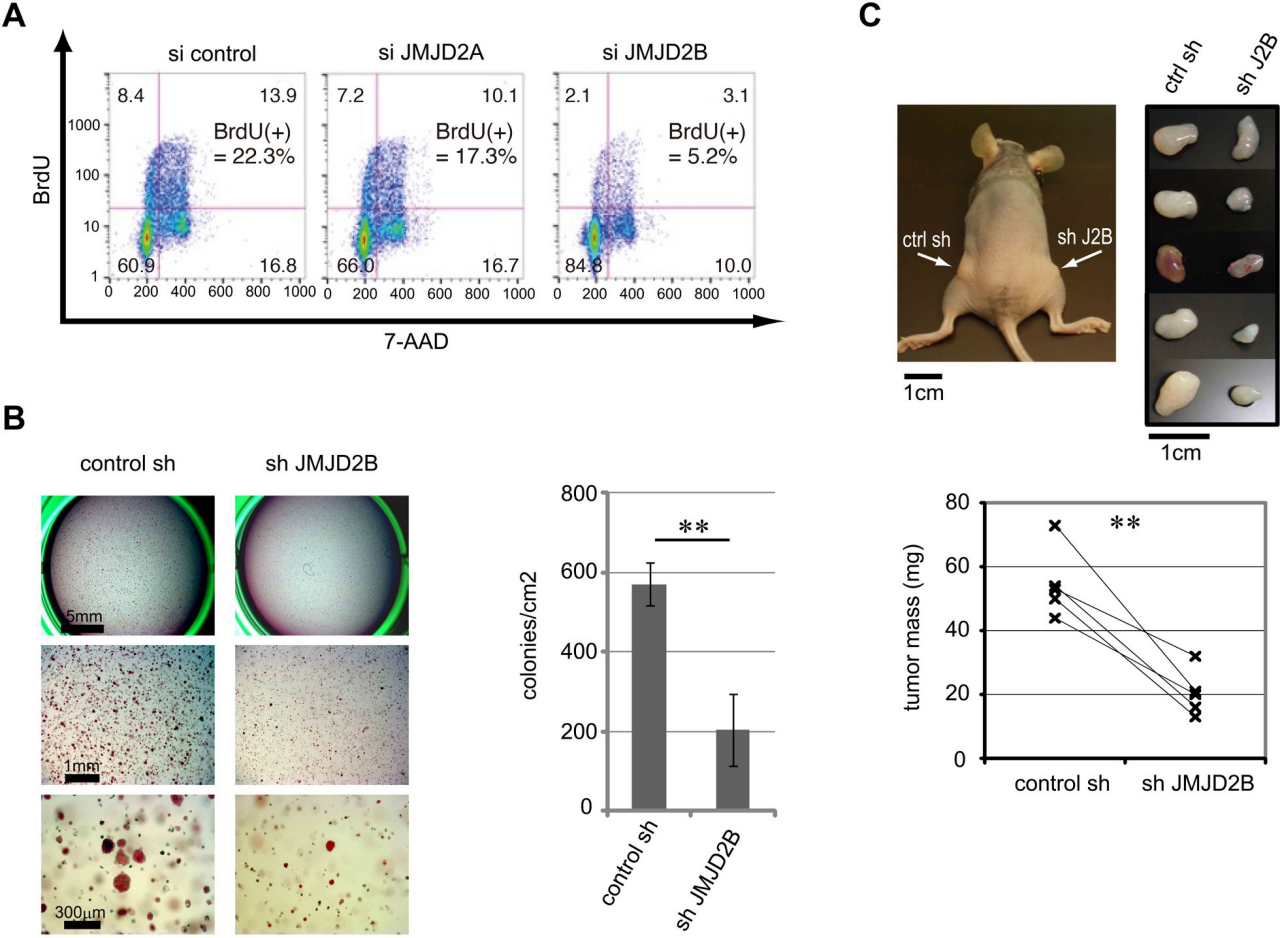
JMJD2B positively regulates the proliferation of ER-positive breast cancer cells. (A) JMJD2B knockdown impairs proliferation. T-47D cells were transfected with either control siRNA or siRNA against JMJD2A or JMJD2B, cultured for 72 hr, pulsed for 1 hr with BrdU, stained with APC-conjugated anti-BrdU antibody and 7-AAD, and analyzed by flow cytometry. Results are representative of four independent trials. (B) JMJD2B knockdown impairs colony formation. Single cell suspensions of ZR-75-1 cells expressing control shRNA or shRNA against JMJD2B were seeded in soft agar. After 14 days, colonies were stained with crystal violet and microscopic fields were photographed (left). The number of colonies/cm2 was determined (right). Data represent the colony density of three wells (mean ± s.d). **p<0.01. Representative data of three independent trials. See also Figure S2. (C) JMJD2B knockdown impairs tumor formation in xenograft model. Upper left: tumors (indicated by arrows) in a representative NIH-III mouse injected with control ZR-75-1 cells on the left flank and JMJD2B-depleted ZR-75-1 cells on the right flank. Upper right: dissected tumors from nude mice. Lower: mass of tumors is shown. **p<0.01.

## Supporting information

S1 File Original data underlying Fig 2A. Data.001: control, Data.002: siJMJD2A, Data.004: siJMJD2B, Original.wsp: original FlowJo gating; Revised-231012.wsp: revised FlowJo gating.
(ZIP)
