# Supplementary figures and images for: Histone Demethylase JMJD2B Functions as a Co-Factor of Estrogen Receptor in Breast Cancer Proliferation and Mammary Gland Development
Source: PLoS One. 2011 Mar 18;6(3):e17830. doi: 10.1371/journal.pone.0017830 (PMC3060874; doi:10.1371/journal.pone.0017830)

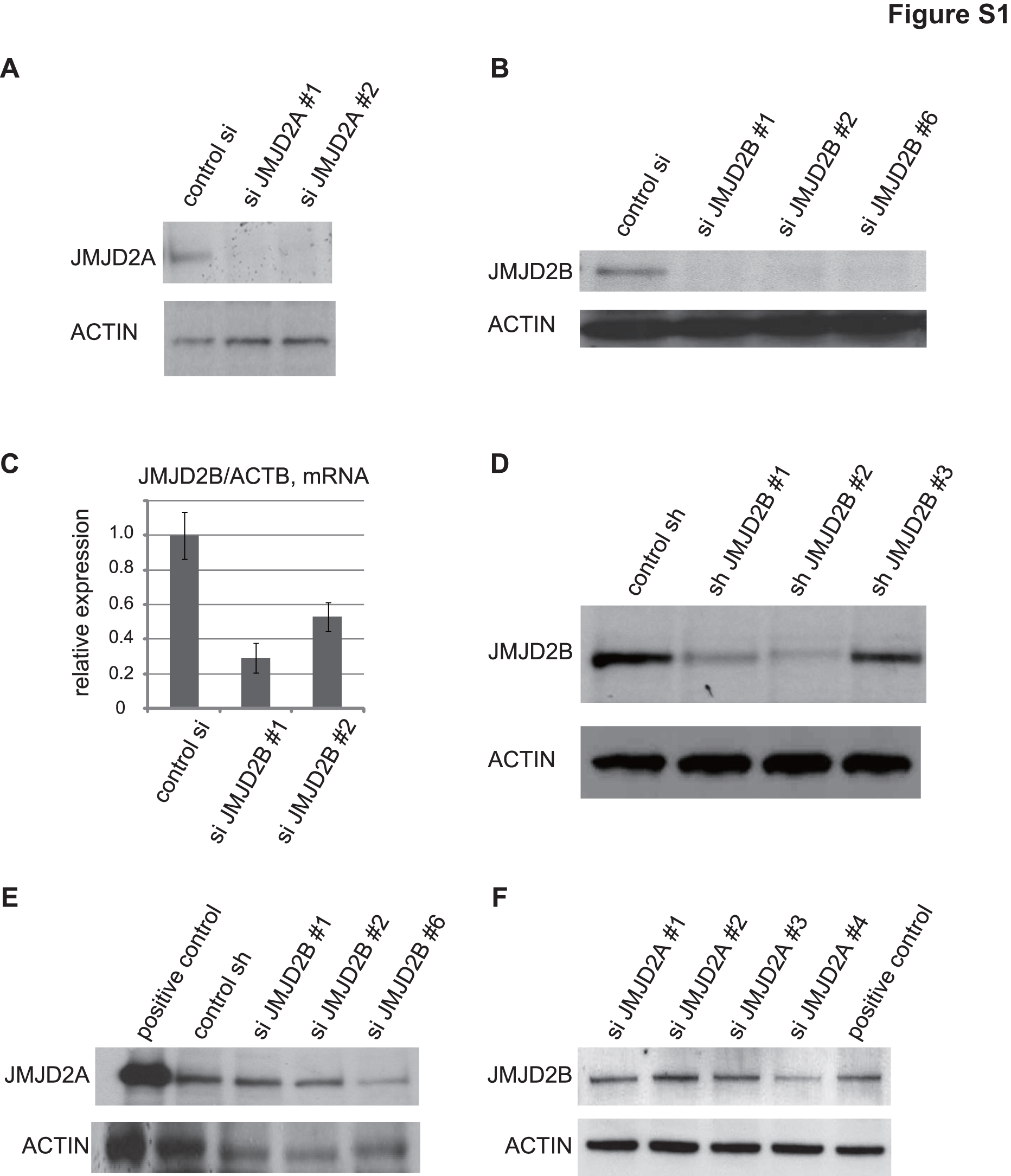

Supplement: Figure S1 — Validation of JMJD2B knockdown efficiency. (A) T-47D cells were transfected with siRNA against JMJD2A (target sequences #1 and #2). (B) T-47D cells were transfected with siRNA against JMJD2B (target sequences #1, #2, and #6). (A and B) Total cell lysate was prepared 72 hr after transfection and subjected to western blotting. (C) T-47D cells were transfected with si JMJD2B #1 or #2. RNA was extracted 72 hr after transfection and subjected to quantitation by real-time RT-PCR. (D) MCF-7 cells were infected with a lentiviral vector harboring JMJD2B shRNA targeting sequence #1, #2 or #3. Total cell lysates were prepared 48 hr after infection and subjected to western blotting. (E) T-47D cells were transfected with siRNA against JMJD2B (target sequences #1, #2, and #6). Total cell lysate was prepared 72 hr after transfection and subjected to western blotting for JMJD2A. The introduction of #6 siRNA reduced JMJD2A protein levels. We therefore used #1 and #2 siRNA in this study. Positive control: protein samples from JMJD2A overexpressed cells. (F) T-47D cells were transfected with siRNA against JMJD2A (target sequences #1, #2, #3 and #4). Total cell lysate was prepared 72 hr after transfection and subjected to western blotting for JMJD2B. (TIF) [file pone.0017830.s001.tif]

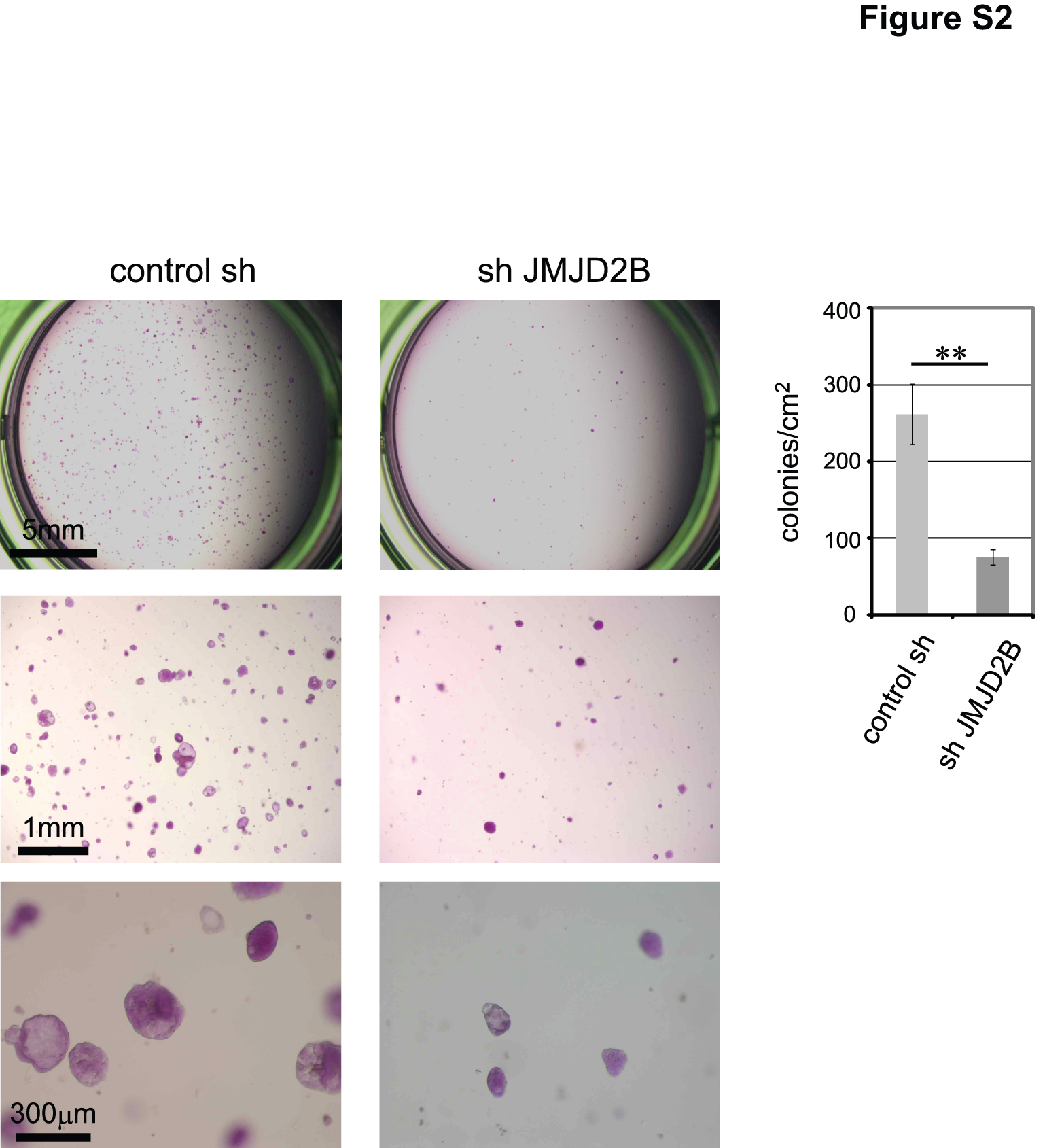

Supplement: Figure S2 — JMJD2B knockdown decreases colony formation of MCF-7 cells. Single cell suspensions of MCF-7 cells expressing control shRNA or shRNA against JMJD2B (target sequence #2) were seeded in 0.35% soft agar. After 14 days, colonies were stained with crystal violet. Microscopic fields were photographed (left). The number of colonies/cm2 was determined (right). Data are the mean number of colonies formed in three wells ± s.d., p<0.01. A representative result from four independent trials. (TIF) [file pone.0017830.s002.tif]

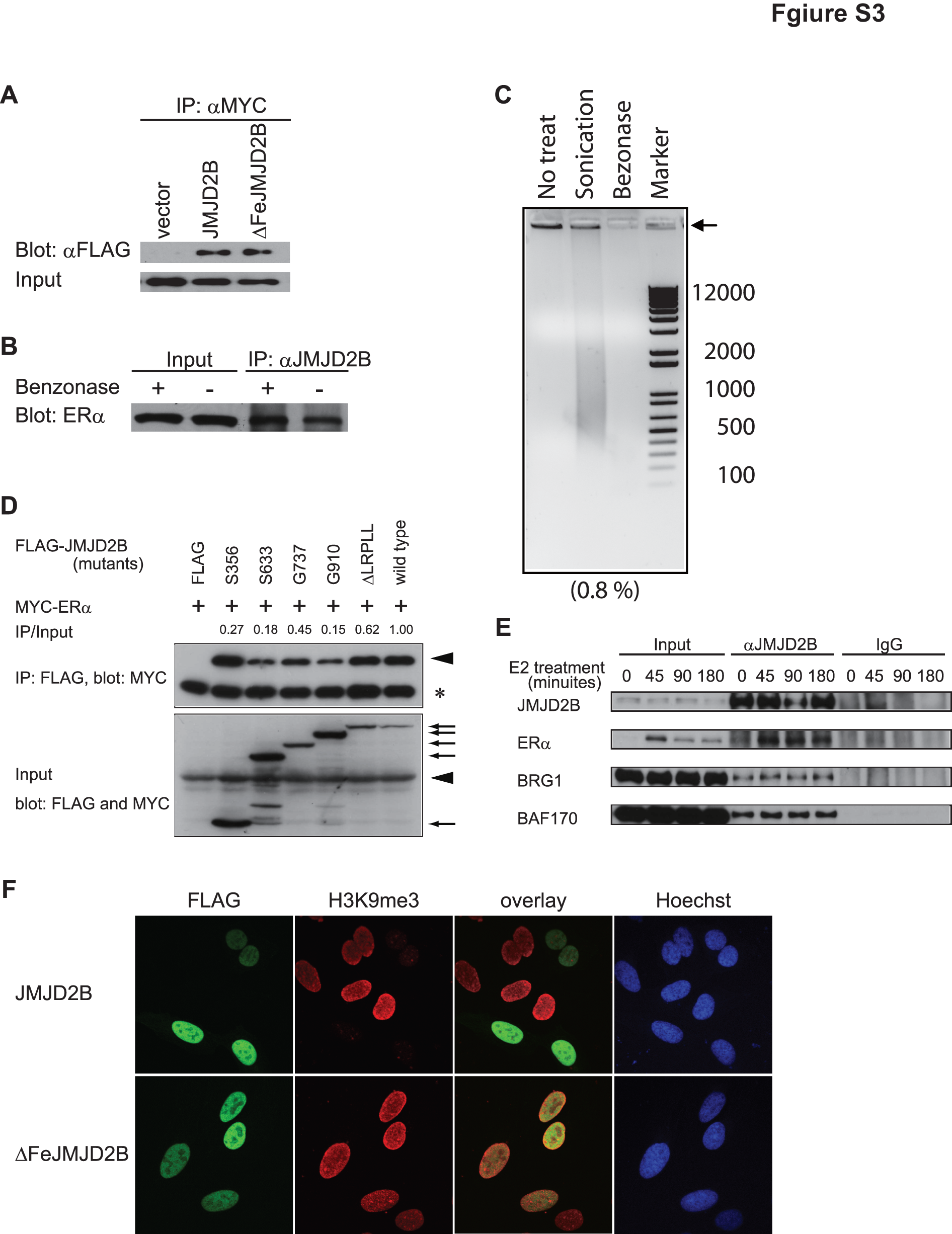

Supplement: Figure S3 — JMJD2B interacts with ERα and SWI/SNF-B complex. (A) Catalytic activity of JMJD2B does not influence the interaction with ER. 293T cells were co-transfected with MYC-ERα and either empty vector, FLAG-JMJD2B or FLAG-ΔFeJMJD2B (a mutant with (H189Y and E191A) point mutations in the iron-binding region). α-MYC immunoprecipitates were analyzed by western blot with α-FLAG antibody. (B) Benzonase treatment does not affect the interaction of endogenous JMJD2B and ER. Nuclear lysates of T-47D cells were lyzed and sonicated in the presence or absence of 500 unit of Benzonase (Novagen) before immunoprecipitation with α-JMJD2B Ab. Input lysate and immunoprecipitated samples were immunoblotted using α-ERα antibody. Full-length blots are presented in Figure S7. (C) Sonication and benzonase treatment digest genomic DNA. Nuclear lysates were treated as in (B). 15% of treated and untreated samples applied for immunoprecipitation were loaded on 0.8% gel. The arrow indicates undigested genomic DNA. (D) The JmjN and JmjC domains are sufficient for co-immunoprecipitation with ERα. 293T cells were co-transfected with MYC-ERα and one of several JMJD2B deletion mutant expression vectors encoding the structures illustrated in Figure 3B. Cell lysates and α-FLAG immunoprecipitates were analyzed by western blot with antibodies against the indicated proteins. Relative intensity of the bands of the Flag-JMJD2B and mutants are shown, normalized to the bands of corresponding input. The values are presented wild type as 1. Arrowheads, ERα bands; arrows, wild type or deletion mutant JMJD2B proteins; asterisk, α-FLAG antibody. (E) Kinetics of Association between JMJD2B and ERα or SWI/SNF-B complex. Nuclear lysates were harvested at indicated time points after E2 stimulation and subjected to immunoprecipitation with control IgG or the antibodies against the indicated proteins. Input lysate and immunoprecipitated samples were then immunoblotted using antibodies against the indicated proteins. (F) JMJD [file pone.0017830.s003.tif]

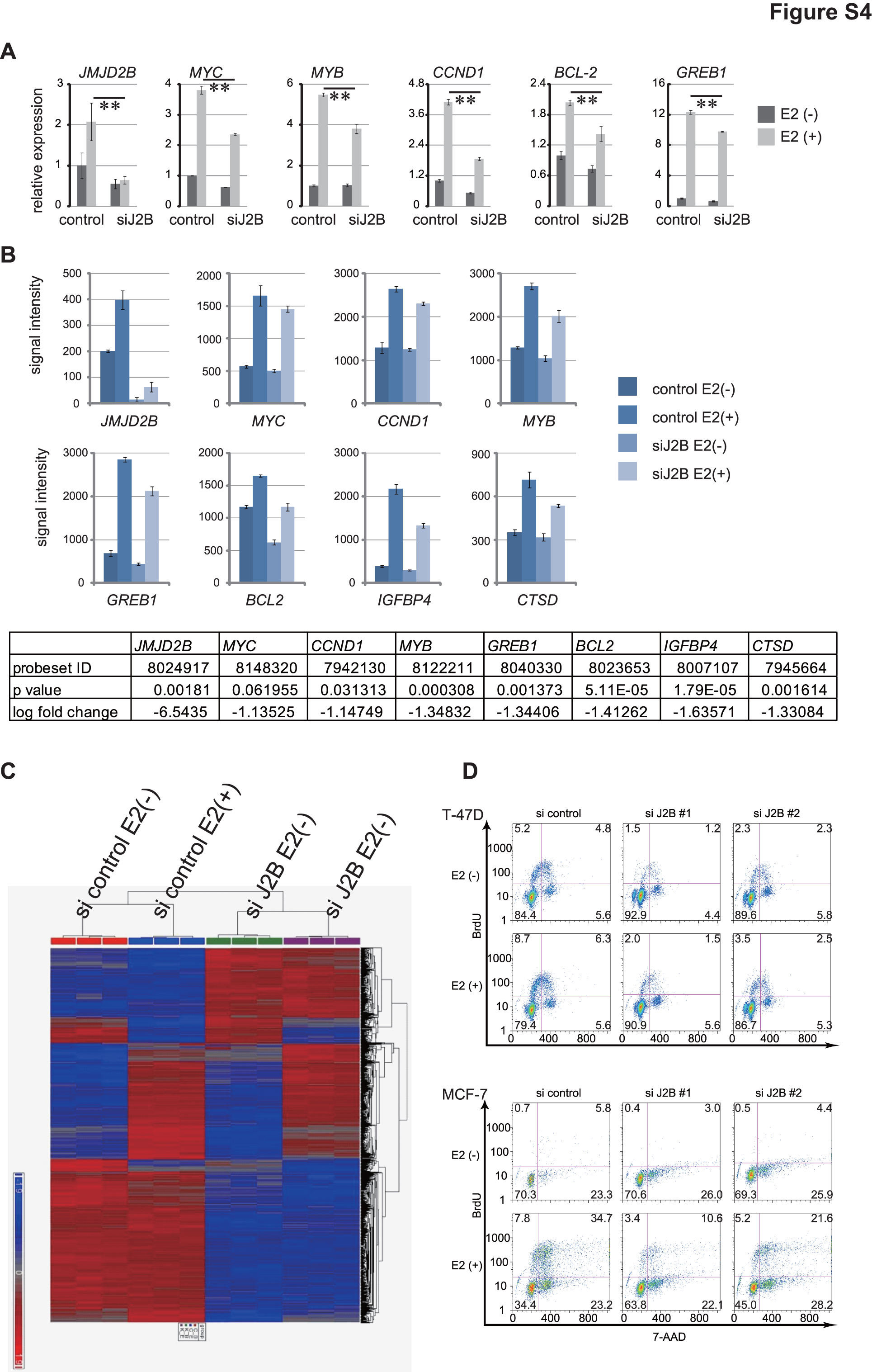

Supplement: Figure S4 — JMJD2B mediates induction of ER target genes and estrogen-dependent proliferation of breast cancer cells. (A) JMJD2B is required for the induction of ER target genes. MCF-7 cells were transfected with either control siRNA or JMJD2B siRNA (target sequence #1), cultured in steroid-free medium for 72 hr, and stimulated with or without E2 for 4 hr. mRNA levels of JMJD2B or the indicated ER target genes were measured by real-time RT-PCR. Results shown are mean mRNA level normalized to the amount of ACTB mRNA ± s.d. of triplicates. **, p<0.01. (B) Microarray data for representative ER target genes. Signal intensities on microarray for representative ER target genes are shown by their mean values (±SD). (C) Heat map representation of differentially expressed genes. One thousand four hundred and thirty-two differentially expressed genes (as calculated using a false discovery rate <0.05 and log-fold change >2) were sorted by hierarchical clustering. Each row represents a gene and each column represents a sample. Red indicates higher expression and blue lower expression. (D) JMJD2B knockdown impairs cellular response to estrogen. T-47D cells or MCF-7 cells transfected with control siRNA or JMJD2B siRNA were cultured in steroid-free medium for 48 hr, stimulated for 24 hr with E2, labeled for 1 hr with BrdU, and stained with anti-BrdU antibody and 7-AAD. The fraction of BrdU-positive cells was determined by flow cytometry. A representative result from three independent experiments. (TIF) [file pone.0017830.s004.tif]

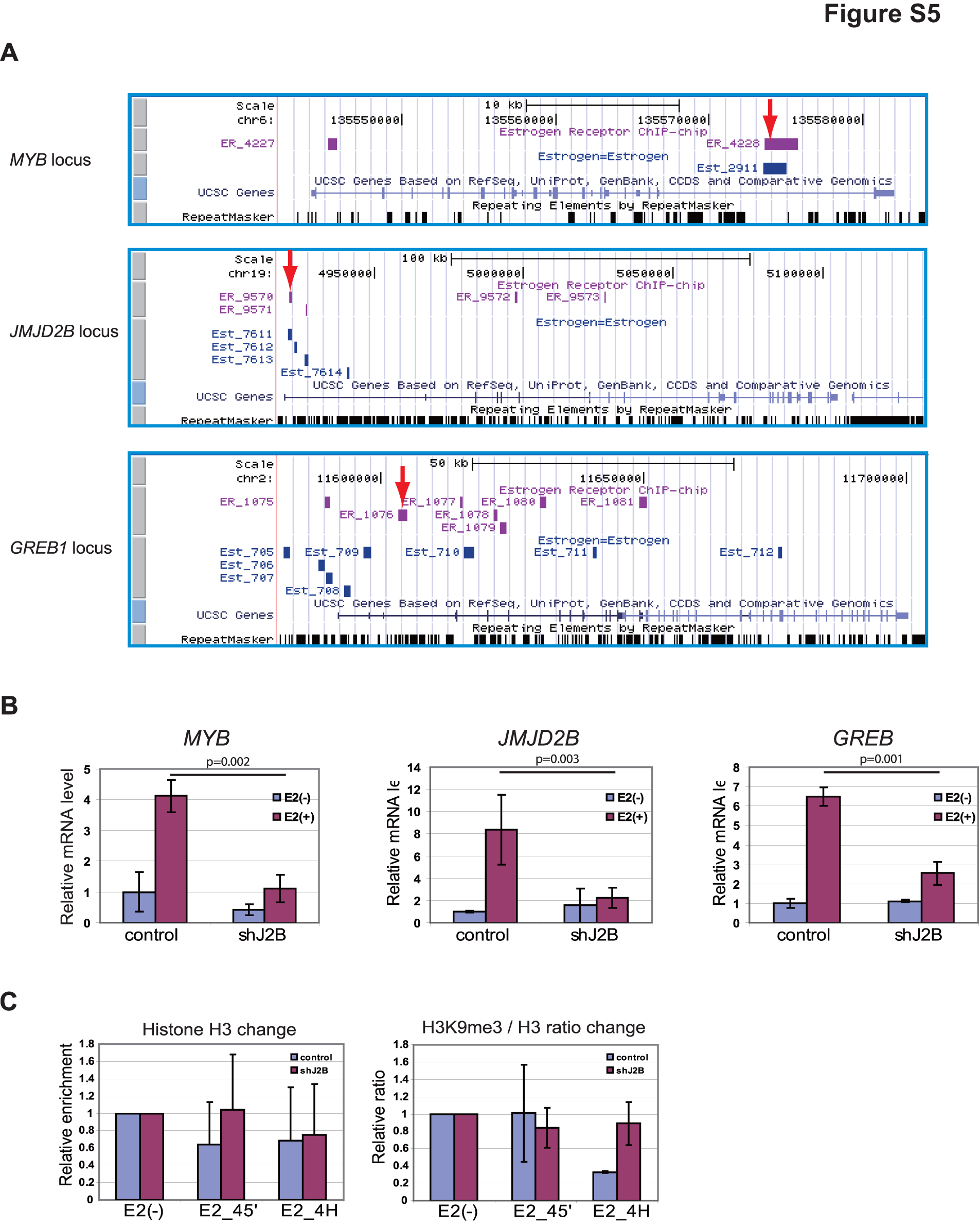

Supplement: Figure S5 — ER binding sites assessed in this study. (A) The UCSC Genome browse map depicts the ER binding sites of MYB locus, JMJD2B locus, and GREB1 locus. Depicted ER binding sites are identified in previously published studies. (B) Effect of JMJD2B knockdown on induction of ER target genes. mRNA levels of JMJD2B or the indicated ER target genes in the corresponding samples applied for the ChIP analysis are shown. (C) The change of H3 and H3K9me3/H3 ratio at Myb ERE. (TIF) [file pone.0017830.s005.tif]

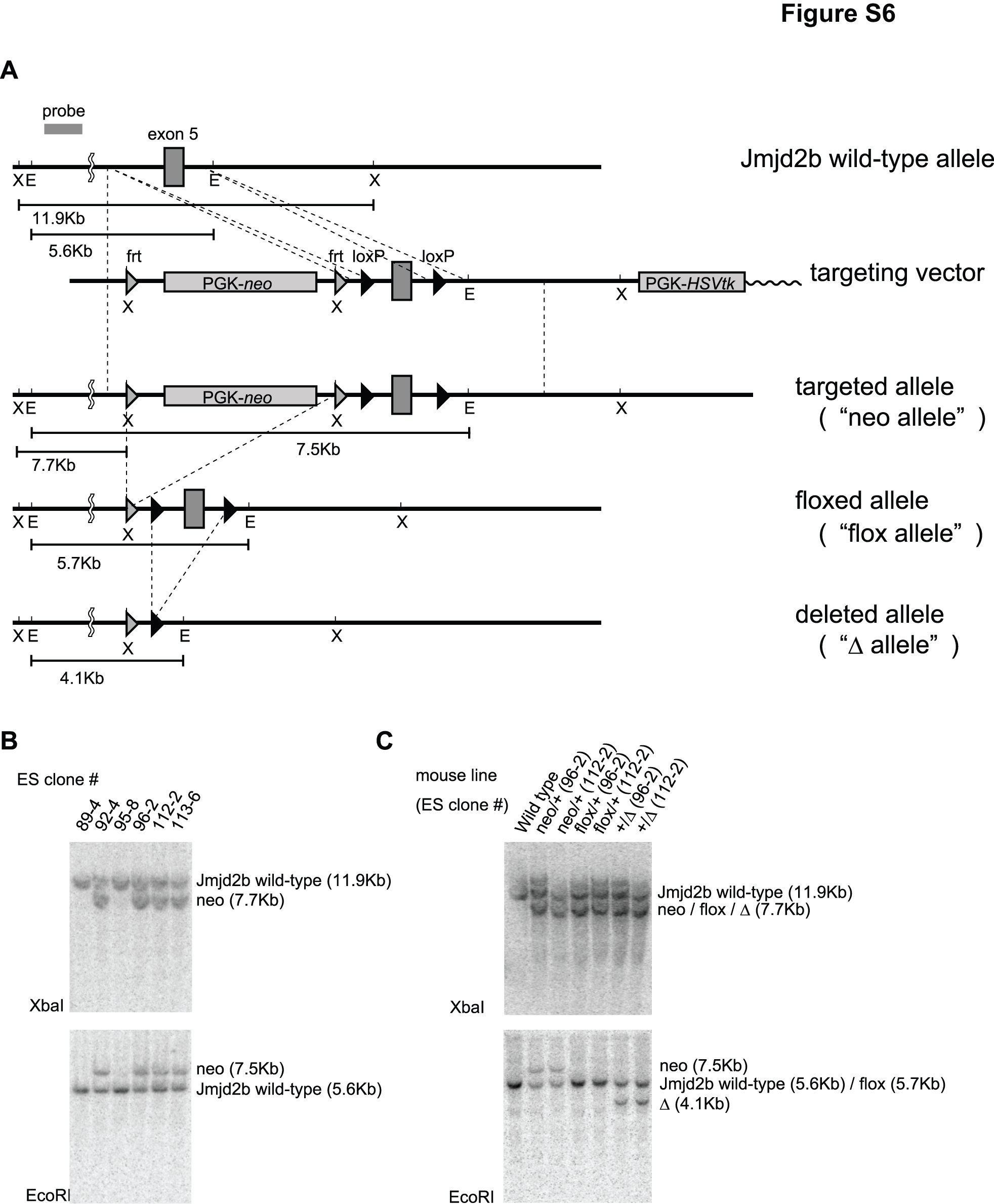

Supplement: Figure S6 — Conditional gene targeting of murine Jmjd2b locus. (A) Introduction of loxP sites into Jmjd2b. A portion of the murine wild type Jmjd2b locus showing exon 5 (grey rectangle) and an 11.9 kb XbaI fragment are shown at the top. The targeting vector was designed to generate floxed exon5 (loxP; black triangles), to flank the PGK-neo cassette with frt sequences (grey triangles), and to introduce a new XbaI site (X). The targeted allele contains a diagnostic 7.7 kb XbaI fragment. The neo cassette was removed in vivo by FLPe recombinase as described in Materials and Methods. Cre-mediated recombination resulted in the deletion of Jmjd2b exon 5. The position of the 5′ flanking probe used for genotyping is indicated. (B) Southern blot analyses to identify Jmjd2b neo/+ ES cells (lanes 2, 4, 5, and 6). Genomic DNA was digested with XbaI (upper panel) or EcoRI (lower panel) and hybridized with the probe indicated in (A). (C) Southern blot analyses to identify Jmjd2b neo/+ mice (lanes 2 and 3), Jmjd2b flox/+ mice (lanes 4 and 5), and Jmjd2b +/Δ mice (lanes 6 and 7). Genomic DNA was digested with XbaI (upper panel) or EcoRI (lower panel) and hybridized with the probe indicated in (A). The numbers in brackets indicate the ES clones from which the mice were derived. (TIF) [file pone.0017830.s006.tif]

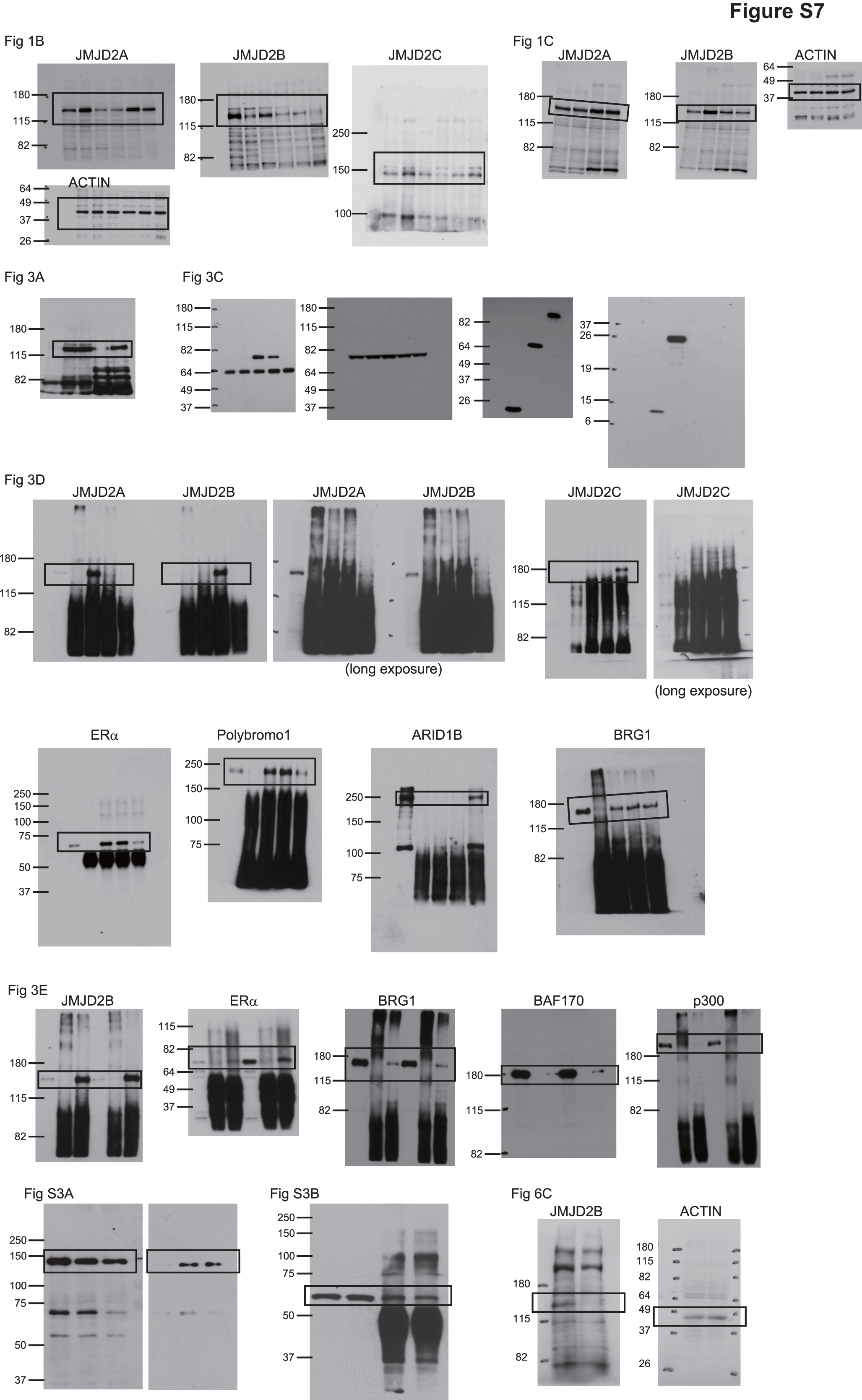

Supplement: Figure S7 — Full-length blots images. Full-length blot images related to Figures 1, 3, S3 and 6 are shown. The cropped regions are indicated. (TIF) [file pone.0017830.s007.tif]
