# Supplementary material for: Histone Demethylase JMJD2B Functions as a Co-Factor of Estrogen Receptor in Breast Cancer Proliferation and Mammary Gland Development
Source: PLoS One. 2011 Mar 18;6(3):e17830. doi: 10.1371/journal.pone.0017830 (PMC3060874; doi:10.1371/journal.pone.0017830)
Supplement: Table S1 — P value of difference in JMJD2B mRNA expression in ER-positive and ER-negative breast cancers in 19 ONCOMINE studies. (DOC) [file pone.0017830.s008.doc]

**Supplementary Table S1. P value of difference in JMJD2B mRNA expression in ER-positive and ER-negative breast cancers in 19 ONCOMINE studies.**

| **Study ID** | **n (ER-)** | **n (ER+)** | **P value** | **Study Name** | **Reference** |
| --- | --- | --- | --- | --- | --- |
| 1 | 77 | 209 | 3.5E-22 | Wang_Breast | 1 |
| 2 | 42 | 57 | 7.3E-17 | Minn_Breast_2 | 2 |
| 3 | 51 | 82 | 8.4E-17 | Hess_Breast | 3 |
| 4 | 64 | 134 | 4.1E-16 | Desmedt_Breast | 4 |
| 5 | 34 | 211 | 6.9E-13 | Ivshina_Breast | 5 |
| 6 | 34 | 213 | 2.9E-12 | Miller_Breast | 6 |
| 7 | 43 | 75 | 1.5E-11 | Chin_Breast | 7 |
| 8 | 11 | 24 | 2.1E-10 | Zhao_Breast | 8 |
| 9 | 28 | 27 | 1.5E-09 | Ginestier_Breast | 9 |
| 10 | 34 | 85 | 2.2E-09 | Sotiriou_Breast_3 | 10 |
| 11 | 60 | 45 | 5.6E-09 | Saal_Breast | 11 |
| 12 | 39 | 57 | 5.0E-08 | Yu-Breast_3 | 12 |
| 13 | 48 | 110 | 7.6E-06 | Bild_Breast | 13 |
| 14 | 24 | 15 | 2.9E-05 | Richardson_Breast_2 | 14 |
| 15 | 9 | 26 | 4.6E-03 | Perou_Breast | 15 |
| 16 | 18 | 56 | 1.1E-02 | Sorlie_Breast | 16 |
| 17 | 7 | 30 | 1.2E-02 | Pollack_Breast_2 | 17 |
| 18 | 3 | 7 | 1.3E-01 | Turashvili_Breast | 18 |
| 19 | 5 | 18 | 6.8E-01 | Ma-Breast | 19 |

****Study name as defined in ONCOMINE.

**Supplementary References, related to Table S1**

1. Wang, Y. et al. Gene-expression profiles to predict distant metastasis of lymph-node-negative primary breast cancer. *Lancet* **365**, 671-9 (2005).

2. Minn, A. J. et al. Genes that mediate breast cancer metastasis to lung. *Nature* **436**, 518-24 (2005).

3. Hess, K. R. et al. Pharmacogenomic predictor of sensitivity to preoperative chemotherapy with paclitaxel and fluorouracil, doxorubicin, and cyclophosphamide in breast cancer. *J Clin Oncol* **24**, 4236-44 (2006).

4. Desmedt, C. et al. Strong time dependence of the 76-gene prognostic signature for node-negative breast cancer patients in the TRANSBIG multicenter independent validation series. *Clin Cancer Res* **13**, 3207-14 (2007).

5. Ivshina, A. V. et al. Genetic reclassification of histologic grade delineates new clinical subtypes of breast cancer. *Cancer Res* **66**, 10292-301 (2006).

6. Miller, L. D. et al. An expression signature for p53 status in human breast cancer predicts mutation status, transcriptional effects, and patient survival. *Proc Natl Acad Sci U S A* **102**, 13550-5 (2005).

7. Chin, K. et al. Genomic and transcriptional aberrations linked to breast cancer pathophysiologies. *Cancer Cell* **10**, 529-41 (2006).

8. Zhao, H. et al. Different gene expression patterns in invasive lobular and ductal carcinomas of the breast. *Mol Biol Cell* **15**, 2523-36 (2004).

9. Ginestier, C. et al. Prognosis and gene expression profiling of 20q13-amplified breast cancers. *Clin Cancer Res* **12**, 4533-44 (2006).

10. Sotiriou, C. et al. Gene expression profiling in breast cancer: understanding the molecular basis of histologic grade to improve prognosis. *J Natl Cancer Inst* **98**, 262-72 (2006).

11. Saal, L. H. et al. Poor prognosis in carcinoma is associated with a gene expression signature of aberrant PTEN tumor suppressor pathway activity. *Proc Natl Acad Sci U S A* **104**, 7564-9 (2007).

12. Yu, K., Ganesan, K., Miller, L. D. & Tan, P. A modular analysis of breast cancer reveals a novel low-grade molecular signature in estrogen receptor-positive tumors. *Clin Cancer Res* **12**, 3288-96 (2006).

13. Bild, A. H. et al. Oncogenic pathway signatures in human cancers as a guide to targeted therapies. *Nature* **439**, 353-7 (2006).

14. Richardson, A. L. et al. X chromosomal abnormalities in basal-like human breast cancer. *Cancer Cell* **9**, 121-32 (2006).

15. Perou, C. M. et al. Molecular portraits of human breast tumours. *Nature* **406**, 747-52 (2000).

16. Sorlie, T. et al. Gene expression patterns of breast carcinomas distinguish tumor subclasses with clinical implications. *Proc Natl Acad Sci U S A* **98**, 10869-74 (2001).

17. Pollack, J. R. et al. Microarray analysis reveals a major direct role of DNA copy number alteration in the transcriptional program of human breast tumors. *Proc Natl Acad Sci U S A* **99**, 12963-8 (2002).

18. Turashvili, G. et al. Novel markers for differentiation of lobular and ductal invasive breast carcinomas by laser microdissection and microarray analysis. *BMC Cancer* **7**, 55 (2007).

19. Ma, X. J. et al. Gene expression profiles of human breast cancer progression. *Proc Natl Acad Sci U S A* **100**, 5974-9 (2003).
