# Supplementary material for: Histone Demethylase JMJD2B Functions as a Co-Factor of Estrogen Receptor in Breast Cancer Proliferation and Mammary Gland Development
Source: PLoS One. 2011 Mar 18;6(3):e17830. doi: 10.1371/journal.pone.0017830 (PMC3060874; doi:10.1371/journal.pone.0017830)
Supplement: Procedure S1 — (DOC) [file pone.0017830.s013.doc]

**Procedures S1**

**Antibodies**

Antibodies recognizing the following proteins were used: JMJD2A (IP; Bethyl, A300-860A, blot; Bethyl, A300-861A), JMJD2B (IP/ChIP; Bethyl, A301-477A, blot; Bethyl, A301-478A), JMJD2C (IP; Bethyl, A300-885A, blot; Life Span, LS-B2856), ACTIN (Sigma-Aldrich, A2066), ERα (ChIP; Santa Cruz, HC-20 (sc-543), blot; Santa Cruz, ER1D5 (sc-73479)), histone H3 (Abcam, ab1791), trimethylated histone H3 lysine 9 (Abcam, ab8898), dimethylated histone H3 lysine 9 (Abcam, ab1220), Polybromo1(IP; Bethyl, A301-590A, blot; Bethyl, A301-591A), ARID1B (Abcam, ab57461), BRG1 (Abcam, ab4081), BAF170 (Santa Cruz, E-6 (sc-17838)), p300 (Santa Cruz, N-15 (sc-584)).

**Primers**

***Primers for real-time RT-PCR***

| **Gene** | **Forward** | **Reverse** |  |
| --- | --- | --- | --- |
| *ACTB* | CATGTACGTTGCTATCCAGGC | CTCCTTAATGTCACGCACGAT |  |
| *JMJD2A* | GGAAGCCACGAGCATCCTATG | GGAACTCTCGAACAGTCATGG |  |
| *JMJD2B* | TACTGTACCCCACGCCATCA | TCAGATTGCCGATGTTCCAC |  |
| *JMJD2C* | CTGTCACCTAGTGCGGAACAA | ATGATGGTTAGGGCAGTGTCT |  |
| *CCND1* | GTGCTGCGAAGTGGAAACC | ATCCAGGTGGCGACGATCT |  |
| *MYC* | CCACAGCAAACCTCCTCACAG | GCAGGATAGTCCTTCCGAGTG |  |
| *MYB* | TCAGGAAACTTCTTCTGCTCACA | AGGTTCCCAGGTACTGCTACA |  |
| *BCL-2* | GGGGAGGATTGTGGCCTTC | CAGGGCGATGTTGTCCACC |  |
| *GREB1* | GGCAGGACCAGCTTCTGA | CTGTTCCCACCACCTTGG |  |

Primers for ChIP

| ER negative | GGGACTCTCGAGGGGATAAG | TGTACCCAAGAACCACGTCA |  |
| --- | --- | --- | --- |
| MYB001 | CACAAACCCAGTCAGCAGAA | GGGGGATTTTAGGGGAGTTT |  |
| MYB002 | ATTCCCAGAGAGGCCCATAG | CAGTGGAGGTCCCTGGTACA |  |
| MYB003 | TCCCATTTTCCACTCTCGTC | AGGGACATGGTGAAGTCTGG |  |
| MYB004 | TTCTACACCCTTCCCCCTTC | GAGGCGCTTTCTTCAGGTAG |  |
| MYB005 | CCTTGTGTGCAGCTTGATGT | TCCCAAAGGAAAGGTTGATG |  |
| MYB006 | TTTGCAACTGGTGTAATGCAG | AAGCCGGGGTGATTAAAACT |  |
| MYB007 | TAGAAAGCGTGCATTTGTGG | CTGAGGAAAGCAGGCAACTC |  |
| MYB008 | GAAGGGCTAAGTGAAGGGAGA | AAGACCAAGTGCCAAATTGC |  |
| MYB009 | TGTCTTGTGCTTTCCTGGTG | GATGACTTGGAACGCCTGAT |  |
| MYB010 | CCTTCCTGGTGTCAACCACT | CTTTCCACAGGATGCAGGTT |  |
| MYB011 | TCACAAGCATCTCCAAAACG | TGGACATACGCAGAAGCAAC |  |
| *JMJD2B* | CACAGTAGACGCTTCCACCA | TCTCCGTGTGTCTGATTGGA |  |
| *GREB1* | AATCAACCACCAAGCCTCAC | CAGATCCCACAAGGGTCACT |  |

**RNA interference**

siRNA target sequences: JMJD2A, GTATGATCTTCCAGACTTA (#1); JMJD2B, GGAAGAACCTCACCTTTGT (#1), GGGAATTCATGATCACATT (#2), and GCGCAGAATCTACCAACTT (#6).

JMJD2B shRNA target sequences: GGAAGGACATGGTCAAGAT (#1) and GGTGGAAGCTGAAATGCGT (#2).

**ChIP assay**

Cells were crosslinked in 1% formaldehyde for 10 min at room temperature, washed in hypotonic lysiss buffer [10 mM Tris-HCl pH7.5, 10 mM NaCl, 3 mM MgCl_2_, 0.5% NP-40, 1 mM PMSF] and lysed in cel lysis buffer [50 mM Tris-Hl pH8.0, 10 mM EDTA, 1% SDS, supplemented with proteinase inhibitor cocktail (Roche)]. Nuclear lysate was sonicated extensively to obtain fragmented chromatin. Two microgram of specific antibodies were coupled to Dynabeads-protein A (Invitrogen) and immunoprecipitation was performed at 4 Cº, over night. Beads were washed with Wash Buffer1 [0.1% SDS, 1% TritonX-100, 2 mM EDTA, 20 mM Tris-HCl (pH8.0), 150 mM NaCl], Wash Buffer2 [0.1% SDS, 1% Triton X-100, 2 mM EDTA, 20 mM Tris-HCl (pH 8.0), 500 mM NaCl], Wash Buffer3 [0.25 M LiCl, 1% NP-40, 1% deoxycholate, 1 mM EDTA, 10 mM Tris HCl (pH 8.0)] and TE. Precipitated DNA was eluted with Elution Buffer [1% SDS and 100 mM NaHCO3], reverse-crosslinked at 65 Cº, purified and subjected to quantitative PCR. Enrichment was indicated by percent fraction of total input DNA. Input DNA was measured for every sample. Refer to Supplementary Information for primer information.

**Gene targeting in mice**

A *Jmjd2b*-targeting vector was constructed in which the *neo* cassette was flanked by *frt* sequences, and *Jmjd2b* exon 5 was flanked by *loxP* sequences (Figure S6A). Cre-mediated removal of exon 5 resulted in a frameshift and translation termination. The conditional targeting vector was used to generate four independent ES cell lines (129/Ola) carrying a floxed *Jmjd2b* exon 5 and a flrted *neo* gene (Figure S6B). The targeted ES clones were injected into blastocysts (C57BL/6). Standard breeding steps were followed to produce animals with the flrted *neo* and floxed *Jmjd2b* alleles (*Jmjd2b*^neo/+^ mice) (Figure S6C). *Jmjd2b*^neo/+^ mice were crossed with mice carrying the *FLPe recombinase* transgene to generate *Jmjd2b*^neo/+^;*FLPe* mice in which the *neo* cassette was excised from the *Jmjd2b* *neo* allele in germ cells. Offspring of *Jmjd2b*^neo/+^;*FLPe* mice carried the *Jmj2b* allele with floxed exon 5 but without the *neo* cassette (*Jmjd2b*^flox/+)^ (Figure S6C). *Jmjd2b*^flox/flox^ mice were born at the expected Mendelian ratio and displayed no abnormalities, indicating that genetic manipulation of the *Jmjd2b* gene did not interfere with essential *Jmjd2b* functions. *Jmjd2b*^flox/+^ mice were then crossed with *Del-Cre* mice carrying the *Cre recombinase* transgene to generate *Jmjd2b*^flox/+^;*Del-Cre* mice in which exon 5 was excised from the *Jmjd2b* floxed allele in germ cells. Offspring of *Jmjd2b*^flox/+^;*Del-Cre* mice carried the *Jmj2b* allele without exon 5 (*Jmjd2b*^+/Δ^) (Figure S6C).

**Microarray analysis**

[1] The transcript assignments are based upon the genome build, UCSC hg19.

[2] After importing CFL files, we used Partek (Partek version 6.5) defaults setting for the subsequent analysis. One-way ANOVA was performed with a 2-fold up -regulated or down-regulated gene expression cutoff. The p value for ANOVA analysis was set as p<0.05 and further adjusted by FDR (Step Up, FDR<0.05). The data have been submitted to GEO (GSE23445).
